# Unveiling the Bioactive Potential of the Aerial Parts of Balkan *Achillea clypeolata*: Comparison with Officinal *Achillea millefolium*

**DOI:** 10.3390/pharmaceutics18050591

**Published:** 2026-05-11

**Authors:** Katarina Šavikin, Aleksandra Jovanović, Andrea Pirković, Ana Alimpić Aradski, Jelena Živković, Tatjana Stević, Antoaneta Trendafilova

**Affiliations:** 1 Institute for Medicinal Plant Research “Dr. Josif Pančić”, Tadeuša Košćuška 1, 11000 Belgrade, Serbia; ksavikin@mocbilja.rs (K.Š.); tstevic@mocbilja.rs (T.S.); 2 Institute for the Application of Nuclear Energy INEP, University of Belgrade, Banatska 31b, 11080 Belgrade, Serbia; ajovanovic@inep.co.rs (A.J.); andrea.pirkovic@inep.co.rs (A.P.); 3 Institute of Botany and Botanical Garden, “Jevremovac”, University of Belgrade-Faculty of Biology, Studentski trg 16, 11000 Belgrade, Serbia; alimpic.ana@bio.bg.ac.rs; 4 Institute of Organic Chemistry with Centre of Phytochemistry, Bulgarian Academy of Sciences, 1113 Sofia, Bulgaria; antoaneta.trendafilova@orgchm.bas.bg; 5 Centre of Competence “Sustainable Utilization of Bio-Resources and Waste of Medicinal and Aromatic Plants for Innovative Bioactive Products” (BIORESOURCES BG), 1000 Sofia, Bulgaria

**Keywords:** chlorogenic acid, UHPLC-MS/MS, skin, anti-inflammatory, collagenase, elastase, hyaluronidase, tyrosinase

## Abstract

**Background/Objectives**: *Achillea millefolium* is a well-known medicinal plant recognized in several pharmacopeias, while the Balkan endemic species *Achillea clypeolata* lacks a pharmacopeial monograph and remains insufficiently studied despite its traditional use. This study aimed to comparatively evaluate the phytochemical composition and biological potential of both species. **Methods**: Chemical composition was studied using UHPLC-MS/MS, HPLC, and FT-IR; anti-inflammatory potential was analyzed by erythrocyte membrane stabilization assay (heat- and hypotonicity-induced hemolysis); and enzyme-inhibitory activity was tested against collagenase, elastase, hyaluronidase, and tyrosinase. In addition, antioxidant activity was evaluated using DPPH, ABTS, and DCFDA assays; antimicrobial activity was determined using the broth microdilution method; and cytotoxic potential was investigated by the MTT assay. **Results**: The major constituents in water–ethanolic extracts were quinic acid derivatives, flavonoids, phenolic acids, and coumarins, with chlorogenic acid, 3,5-dicaffeoylquinic acid, cosmosiin, cynaroside, rutin, and hyperoside as dominant in both species. Extracts exhibited marked anti-inflammatory activity, where *A. millefolium* provided greater protection under heat-induced hemolysis, and both extracts showed comparable efficacy under osmotic stress. Concentration-dependent inhibition of collagenase, elastase, hyaluronidase, and tyrosinase (concentration from 62.5 to 1000 µg/mL), along with significant antioxidant activity in ABTS and DPPH assays, was observed. In MRC-5 cells, the extracts reduced AAPH-induced ROS levels up to 50 µg/mL, while higher concentrations showed diminished effects. Moderate cytotoxicity was observed, with *A. clypeolata* displaying stronger effects at 50–100 µg/mL. Both *Achillea* species exhibited broad-spectrum antimicrobial activity, with pronounced effects against Gram-positive bacteria. **Conclusions**: The results support the traditional use of *Achillea* species and highlight *A. clypeolata* as a promising, yet underexplored, source of bioactive compounds for dermatological and pharmaceutical applications.

## 1. Introduction

The genus *Achillea* (Asteraceae) comprises more than 130 species [1], many of which have a long history of use in traditional medicine, particularly for wound healing and skin-related disorders [2,3]. *Achillea millefolium* L. (common yarrow) (AM) is the most extensively studied species, officially recognized in several pharmacopeias. Its topical application is associated with anti-inflammatory, antioxidant, and wound-healing effects, largely attributed to its phenolic acids, flavonoids, sesquiterpene lactones, and essential oil [4]. According to the European Medicines Agency (EMA) [5], the *A. millefolium* herb is indicated for a temporary loss of appetite, symptomatic treatment of mild, spasmodic gastrointestinal complaints including bloating and flatulence, symptomatic treatment of minor spasms associated with menstrual periods, and treatment of small superficial wounds. In traditional medicine, yarrow is widely used. Previous ethnomedicinal studies have suggested its use for loss of appetite, stomach disorders, diarrhea, liver problems, cough and cold, renal complaints, circulation improvement, hemorrhoids, palpitations, and wounds, as well as menstrual complaints, cramps, and inflammation of the skin and mucous membranes [6,7,8]. A recent study by Dabbaghi et al. suggests that species of the genus *Achillea* may serve as potential agents for ameliorating different types of toxicity [1].

In contrast, Balkan endemic species *Achillea clypeolata* Sm. (AC), commonly known as Balkan yarrow or moonshine, has no pharmacopeial monograph and remains poorly investigated despite traditional evidence suggesting its use for similar purposes [9]. Previous phytochemical studies have revealed notable concentrations of polyphenolic compounds and terpenoids, as well as essential oil, implying that this species may exert bioactivities relevant to skin health [10,11]. Moreover, Aćimović et al. predicted high anti-inflammatory potential of *A. clypeolata* through in silico analysis and noted that activity may be highly diverse depending on the active compounds in the species [11]. Nevertheless, there is a lack of systematic comparative studies that evaluate its potential alongside the officinal species *A. millefolium*.

Oxidative stress, chronic low-grade inflammation, and the activity of enzymes such as collagenase, elastase, hyaluronidase, and tyrosinase are involved in a wide range of biological processes, including skin aging. Natural products capable of modulating these pathways are increasingly sought as safer alternatives or complementary agents in both biomedical and dermatological applications [12,13].

Given the increasing demand for natural agents that modulate inflammation, oxidative stress, and enzymatic processes associated with skin aging, a comparative evaluation of the aerial flowering parts of *A. clypeolata* and the officinal species *A. millefolium* is of particular importance. To our knowledge, although both species have been studied, no research has yet examined their extracts prepared and analyzed under identical conditions using the same methods.

We hypothesized that *A. clypeolata* possesses a phytochemical profile and biological activities comparable to, or potentially distinct from, those of *A. millefolium*, which could support its consideration as a promising natural source for skin-care applications. Therefore, the present study aimed to: (i) characterize and compare the chemical composition of *A. clypeolata* and *A. millefolium*; (ii) evaluate their anti-inflammatory, antioxidant, antimicrobial, and cytotoxic potential; and (iii) assess their inhibitory activity against key skin-aging-related enzymes, such as collagenase, elastase, hyaluronidase, and tyrosinase.

## 2. Materials and Methods

### 2.1. Plant Material

The dried aerial parts (upper 20 cm) of *Achillea millefolium* (AM) were obtained from the Production Sector of the Institute for Medicinal Plants Research “Dr Josif Pančić”, Belgrade, Serbia (control number: 35250824). The aerial parts (upper 20 cm) of *Achillea clypeolata* (AC) were collected from their natural habitat on Stara planina (Serbia) during the full flowering stage in July 2024. The climate of the collection area is classified as moderate-continental, while the predominant soils in Serbian mountainous regions are Cambisols (brown forest soils), with an occasional occurrence of rendzinas on limestone substrates. Voucher specimens (Nos. 0724AMJP and 0724ACSP) were deposited in the herbarium of the Institute for Medicinal Plants Research “Dr Josif Pančić”, Belgrade, Serbia. The plant material was dried, pulverized by the laboratory mill (IKA A11 basic, Staufen, Germany), and sieved through a set of sieves standardized according to the Yugoslav Pharmacopoeia 2000 [14]. For the extraction, a particle size fraction of 0.75 to 2 mm was used.

### 2.2. Preparation of Extracts

Liquid extracts were obtained by digestion (2 times for 6 h) on a hot plate at 35 °C using 70% ethanol (EtOH), with a solid-to-liquid ratio of 1:20, and further evaporated under vacuum (BUCHI Rotavapor R-114, BÜCHI Labortechnik AG, Flawil, Switzerland) until the residual EtOH concentration was below 5%. The process was conducted at a water bath temperature of 50 °C, according to the manufacturer’s recommendation. Vacuum and rotation speed were maintained under standard laboratory conditions routinely applied for solvent evaporation and kept constant for both samples. To obtain dry extracts, they were subjected to lyophilization (lyophilizer Christ Beta-2-8 LD plus, Martin Christ, Osterode am Harz, Germany). The dry residue yield of the obtained extracts was 4.91% for *A. clypeolata* and 4.65% for *A. millefolium*.

### 2.3. UHPLC-MS/MS Analysis

UHPLC-MS/MS analysis was performed on a high-resolution Q Exactive Plus^®^ hybrid quadropole-Orbitrap^®^ mass spectrometer (Thermo Fisher Scientific, Bremen, Germany) equipped with a heated electrospray ionization source, coupled with a Vanquish UHPLC system (Thermo Fisher-Scientific, Bremen, Germany). Chromatographic separation was carried out on an Accucore^TM^ C18 (Thermo Fisher-Scientific, Bremen, Germany) analytical column (150 × 2.1 mm, 2.6 µm) using 0.1% HCOOH in H_2_O (A) and 0.1% HCOOH in CH_3_CN (B) in a gradient mode as described by Angelova et al. [15].

### 2.4. HPLC-PDA Analysis of Caffeoylquinic Acids

The HPLC analysis was carried out on a Shimadzu Nexera-I-LC-2040C 3D Plus liquid chromatograph equipped with a photodiode array detector (Shimadzu, Tokyo, Japan) on an analytical column Force C18 (150 × 4.6 mm, 3 µm, Restek Corporation, Bellefonte, PA, USA). The elution was performed in a gradient mode using 0.1% HCOOH in H_2_O (A) and methanol (B) as described by Ivanova et al. [16]. Chlorogenic acid (5-CQA) and 3,4-, 1,5-, 3,5-, and 4,5-dicaffeoylquinic acids (DCQAs) purchased from Phytolab GmbH & Co. KG (Vestenbergsgreuth, Germany) were used as standards for the calibration curves. The experiments were performed in triplicate, and the results were expressed as mg/g DE (dry extract).

### 2.5. HPLC Analysis of Flavonoids

HPLC measurements were carried out using an Agilent 1260 RR system (Agilent, Waldbronn, Germany) fitted with a diode array detector with a spectral range of 190–550 nm according to a previously published procedure [17]. Chromatographic separation was achieved on a Zorbax SB-C18 reversed-phase column (150 × 4.6 mm, 5 μm; Agilent, Waldbronn, Germany). The mobile phase consisted of solvent A (1% *v*/*v* orthophosphoric acid in water) and solvent B (acetonitrile). Elution was performed under gradient conditions as follows: 90–85% A from 0 to 2.6 min; 85% A from 2.6 to 8 min; 85–80% A from 8 to 10.8 min; 80% A from 10.8 to 18 min; 80–70% A from 18 to 23 min; 70–50% A from 23 to 25 min; 50–30% A from 25 to 27 min; 30–10% A from 27 to 29 min; 10–0% A from 29 to 31 min, followed by 0% A until 34 min. The flow rate was set to 0.8 mL/min, the injection volume was 8 μL, and the column temperature was maintained at 40 °C. Detection was conducted at 260, 280, 320, and 360 nm. Compound identification was based on a comparison of retention times and UV spectra with those of reference standard compounds purchased from ChemFaces (Wuhan, Hubei, China). Quantitative analysis was performed using external calibration curves; the experiments were performed in triplicate, and results were expressed as milligrams per gram of dry extract (mg/g DE).

### 2.6. FT-IR Analysis

The *A. millefolium* (AM) and *A. clypeolata* (AC) extracts were analyzed using Fourier transform infrared spectroscopy (FT-IR). The spectra were obtained by the ATR technique in a Nicolet IS35 FTIR-ATR spectrometer (Thermo Fisher Scientific, Uppsala, Sweden) in the absorption range between 500 cm^−1^ and 4000 cm^−1^.

### 2.7. The Assessment of the Enzyme Inhibition Capacity of the Extracts

#### 2.7.1. Sample and Positive Control Dilutions

The crude ethanolic extracts from *A. millefolium* and *A. clypeolata* leaves were diluted in 70% ethanol using a twofold serial dilution from 62.5 µg/mL to 1000 µg/mL. Positive controls for the enzyme inhibition assays included ethylenediaminetetraacetic acid (EDTA) (6.25–100 µg/mL), kojic acid (15.625–1000 µg/mL), and ascorbic acid (7.81–125 µg/mL), which were dissolved in distilled water, while epigallocatechin gallate (EGCG) (3.82–125 µg/mL) and oleanolic acid (7.81–125 µg/mL) were dissolved in dimethyl sulfoxide (DMSO).

#### 2.7.2. Collagenase Inhibition Assay

The inhibitory effect of *A. millefolium* and *A. clypeolata* leaf ethanolic extracts against collagenase was measured in triplicate using a slightly modified protocol described by Wittenauer et al. [18]. Collagenase (1.1 U/mL) and the synthetic substrate FALGPA (N-(3-[2-furyl]acryloyl)-Leu-Gly-Pro-Ala) (1 mM) were prepared in 50 mM Tricine buffer containing 400 mM sodium chloride and 10 mM calcium chloride (pH 7.5). First, the reaction mixture containing the sample (*S*) was prepared by mixing 30 μL of the extract or standard (EDTA) at different concentrations with 10 μL of enzyme solution and 60 μL of Tricine buffer. Subsequently, the mixture was incubated for 20 min at 37 °C. Then, 20 μL of the 1 mM FALGPA solution was added to initiate the reaction. Incubation continued for another 20 min at 37 °C. For the control (*C*), distilled water was added instead of the sample solution. The color control (*CC*) for each sample contained only the sample at different concentrations and the buffer. EDTA at different concentrations was used as a positive control. The change in absorbance was measured on a Multiskan SkyHigh Microplate Spectrophotometer (Thermo Fisher Scientific, Waltham, MA, USA) at 335 nm for 20 min after the reaction started. The relative inhibition of collagenase was calculated using the following formula (Equation (1)):

(1) $$Collagenase\;inhibitory\;activity\;rate\;(\%)\;=\;\frac{C-(S-CC)}{C}\;\times \;100\%$$

Based on the inhibition values obtained at different concentrations, a dose–response curve was constructed, and the IC_50_ (the concentration required to inhibit an enzyme function by 50%) values (µg/mL) were determined.

#### 2.7.3. Elastase Inhibition Assay

The inhibition rate of porcine pancreatic elastase by *A. millefolium* and *A. clypeolata* leaf ethanolic extracts was determined in triplicate spectrophotometrically using a modified protocol previously published by Lee et al. [19]. The reaction mixture (*S*) contained 120 µL of 0.2 M Tris–HCl buffer (pH 8.0), 20 µL of 10 µg/mL elastase, and 40 µL of the sample (extract or standard in various concentrations). The control (*C*) contained distilled water instead of the sample, while EGCG and oleanolic acid, dissolved at different concentrations, served as positive controls. After pre-incubation for 20 min at 25 °C, 20 µL of substrate SANA (N-succinyl-Ala-Ala-Ala-*p*-nitroanilide) (8 mM) was added to each well to start the reaction. The change in absorbance of *p*-nitroaniline was measured at 410 nm after incubation for 20 min at 25 °C. A color control (*CC*) was prepared for each sample, containing only the sample at different concentrations and the buffer. The elastase inhibitory activity of the samples was calculated using the following formula (Equation (2)):

(2) $$Elastase\;inhibitory\;activity\;rate\;(\%)\;=\;\frac{C-(S-CC)}{C}\;\times \;100\%$$

Based on inhibition values across different concentrations, a dose–response curve was constructed, and the IC_50_ (µg/mL) was determined.

#### 2.7.4. Hyaluronidase Inhibition Assay

Anti-hyaluronidase activity of *A. millefolium* and *A. clypeolata* leaf ethanolic extracts was determined in triplicate spectrophotometrically by measuring the amount of N-acetylglucosamine formed from sodium hyaluronate, following a slightly modified protocol described by Liyanaarachchi et al. [20]. A solution of hyaluronidase isolated from bovine testes (10 μL), prepared in 0.1 M acetate buffer (pH 3.5) at a concentration of 4200 U/mL, was mixed with 50 μL of the sample (*S*) diluted to various concentrations and incubated at 37 °C for 20 min. Then, 20 μL of 12.5 mM calcium chloride was added to the reaction mixture and incubated at 37 °C for 10 min. This Ca^2+^-activated hyaluronidase was then mixed with 50 μL of sodium hyaluronate (1.2 mg/mL) dissolved in 0.1 M acetate buffer (pH 3.5). After incubation at 37 °C for 40 min, 10 μL of 0.9 M sodium hydroxide and 20 μL of 0.2 M potassium tetraborate tetrahydrate were added, followed by incubation in boiling water for 3 min. After cooling to room temperature, 50 μL *p*-DMAB solution (*p*-dimethyl-aminobenzaldehyde) (0.25 g *p*-DMAB dissolved in 21.88 mL of 100% acetic acid and 3.12 mL of 10 N hydrochloric acid) was added and incubated at 37 °C for 10 min. The control (*C*) contained distilled water instead of the sample, while oleanolic acid and ascorbic acid at different concentrations were used as references. A color control (*CC*) was prepared for each sample, containing only the sample at different concentrations and the buffer. Absorbance was measured at 585 nm. The percentage of hyaluronidase inhibition was calculated using Equation (3):

(3) $$Hyaluronidase\;inhibition\;activity\;rate\;(\%)\;=\;\frac{C-(S-CC)}{C}\;\times \;100\%$$

Based on inhibition values across different concentrations, a dose–response curve was constructed, and the IC_50_ (µg/mL) was determined.

#### 2.7.5. Tyrosinase Inhibition Assay

The inhibition of tyrosinase activity by the *A. millefolium* and *A. clypeolata* leaf ethanolic extracts was determined in triplicate spectrophotometrically in 96-well microtiter plates using a slightly modified method of Masuda et al. [21]. The test sample (*S*) contained 80 μL of sodium phosphate buffer (0.1 M, pH 7.0), 40 μL of tyrosinase, and 40 μL of the sample at various concentrations. The control (*C*) contained 120 μL of sodium phosphate buffer (0.1 M, pH 7.0) and 40 μL of tyrosinase (46 U/mL) dissolved in the same buffer. Kojic acid dissolved in buffer at different concentrations was used as a reference. After adding 40 μL of L-DOPA (2.5 mM) to the wells of the control and reaction mixtures and incubating at 25 °C for 30 min, absorbance values were measured at 475 nm. The color control (*CC*) prepared for each sample contained only the sample at different concentrations and the buffer. The percentage of tyrosinase inhibition was determined using the following formula (Equation (4)):

(4) $$Tyrosinase\;inhibition\;activity\;rate\;\left(\%\right)=\;\frac{C-\left(S-CC\right)}{C}\;\times \;100\%\;$$

Based on inhibition values across different concentrations, a dose–response curve was constructed, and the IC_50_ (µg/mL) was determined.

### 2.8. Cytotoxicity and Antioxidant Cell Assays

#### 2.8.1. Cell Culture

Human fetal lung fibroblasts (MRC-5) were propagated in 25 cm^2^ tissue culture flasks in a humidified incubator with 5% CO_2_ at 37 °C in a complete medium containing RPMI 1640 (Biowest, Nuaillé, France), 10% fetal calf serum (FCS, Gibco, Waltham, MA, USA), and 1% antibiotic-antimycotic solution (Capricorn Scientific GmbH, Ebsdorfergrund, Germany). After reaching 70% confluence, the cells were trypsinized (0.25% trypsin, Biowest, Nuaillé, France), seeded in 96-well plates (1.5 × 10^4^ cells/well), and left to attach to wells for 24 h at 37 °C (5% CO_2_).

#### 2.8.2. Treatments Preparation

Stock solutions of the AM and AC extracts were prepared in DMSO (Sigma Aldrich, St. Louis, MO, USA) at a concentration of 100 mg/mL and kept at 4 °C. For the experiment, final concentrations of each extract were prepared from the stock solution by dissolving into fresh complete cell medium to reach final concentrations of 6.25, 12.5, 25, 50, and 100 µg/mL. These concentrations were further used for cell treatments.

#### 2.8.3. Cytotoxicity Evaluation

The MRC-5 cells were treated with various concentrations of AM and AC extracts prepared as described in Section 2.8.2. Treatments preparation, in a total volume of 100 µL per well. Following the incubation with the treatments or medium alone (control) at 37 °C for 24 h, an MTT assay was performed. MTT reagent (thiazolyl blue tetrazolium bromide, 1 mg/mL, Sigma Aldrich, St. Louis, MO, USA) was added (10 µL per well), and the cells were left for 3 h in the dark at 37 °C for the reaction to occur. Further, purple formazan crystals were dissolved by adding the sodium dodecyl sulfate (10% SDS in 0.01 M HCl, Sigma Aldrich, St. Louis, MO, USA) in a volume of 100 µL per well. The absorbance was measured at 570 nm on a microplate reader (BioTek ELx800, Winooski, VT, USA) after the complete solubilization of the crystals. Three independent experiments, each performed in duplicate (*n* = 6), were conducted.

#### 2.8.4. H2DCFDA Assay (2′,7′-Dichlorofluorescin Diacetate)

To evaluate the ROS (reactive oxygen species) production in MRC-5 cells, we followed the manufacturer’s instructions for the DCFDA assay. Specifically, 5 μM of the cell-permeable oxidation-sensitive probe, H2DCFDA (Merck Millipore, Darmstadt, Germany, 2′,7′-dichlorofluorescin diacetate), using phosphate-buffered saline (PBS, Sigma Aldrich, Darmstadt, Germany) as the diluent, was added to cells and left for 45 min in the dark. Next, the cells were washed with PBS, and the treatments with various concentrations of extracts were added to the cells together with 25 mM AAPH (2,2′-azobis(2-methylpropionamidine) dihydrochloride, Merck Millipore, Darmstadt, Germany) as a free radical initiator, in a total volume of 100 μL/well. After 1 h of incubation, treatments were removed, and cells were rinsed with PBS. Due to the conversion of non-fluorescent H2DCFDA to the highly fluorescent 2′,7′-dichloro fluorescein (DCF), the intracellular ROS generation level in cells was determined by measuring the fluorescence at 485/535 nm using a fluorescent microplate reader (Wallac 1420 multilabel counter Victor 3V, PerkinElmer Inc., Waltham, MA, USA). Data were expressed as relative fluorescence intensity. Three independent experiments, each performed in duplicate (*n* = 6), were conducted.

### 2.9. Measurement of Free Radical Neutralization Capacity of the Extracts

The antioxidant activity of AM and AC extracts was also evaluated using two complementary in vitro spectrophotometric assays: the ABTS and DPPH methods.

#### 2.9.1. ABTS Assay

The ABTS^•+^ radical cation decolorization method was used to assess the free radical scavenging capacity of the extracts [22]. A mixture of ABTS solution (5 mL of water containing 0.019 g of ABTS, Sigma-Aldrich, St. Louis, MO, USA) and potassium persulfate solution (88 µL, Centrohem d.o.o., Stara Pazova, Serbia) was allowed to react for 24 h at 4 °C. The resulting ABTS^•+^ working solution was diluted with 96% ethanol (Centrohem d.o.o., Stara Pazova, Serbia) to achieve an absorbance of approximately 0.700 at 734 nm. Then, 2 mL of the ABTS^•+^ solution was combined with 20 µL of extract or control at different concentrations, and after 6 min of incubation, the absorbance was measured at 734 nm. The percentage of radical neutralization was calculated as follows (Equation (5)):

(5) $$\%\;neutralization\;=\;\frac{A_{ABTS\;}-\;A_{x}\;}{A_{x}}\;\times \;100$$

where *A_ABTS_* is the absorbance of the ABTS solution in ethanol containing the extraction medium, and *A_x_* corresponds to the absorbance measured after adding the extract. Ascorbic acid (Sigma-Aldrich, St. Louis, MO, USA) was used as a positive control. Antioxidant activity was expressed as IC_50_ (µg/mL), representing the extract concentration required to neutralize 50% of the ABTS radicals. IC_50_ values were determined from the regression analysis of percent inhibition versus extract concentration. All measurements were performed in triplicate.

#### 2.9.2. DPPH Assay

The DPPH radical scavenging assay was performed similarly to evaluate the antioxidant potential of the extracts [22]. An ethanol DPPH^•^ solution was prepared by dissolving DPPH powder (Sigma-Aldrich, St. Louis, MO, USA) in 96% ethanol, adjusting to an absorbance of approximately 0.800 at 517 nm. Various concentrations of extract or control (200 μL) were mixed with 2 mL of ethanol DPPH^•^ solution. After 20 min of incubation, the absorbance was recorded at 517 nm. The percentage of inhibition was calculated using Equation (6):

(6) $$\%\;neutralization\;=\;\frac{A_{DPPH\;}-\;A_{x}\;}{A_{x}}\;\times \;100$$

where *A_DPPH_* is the absorbance of the ethanolic DPPH solution with the extraction medium, and *A_x_* is the absorbance after adding the extract. Ascorbic acid was used as a positive control. Antioxidant activity was expressed as IC_50_ (µg/mL), corresponding to the concentration needed to scavenge 50% of DPPH radicals. IC_50_ values were obtained from concentration–response curves, and measurements were carried out in triplicate.

### 2.10. Photoprotective Activity Assay

The photoprotective effect of AM and AC extracts was assessed using a previously described in vitro method [23]. The sun protection factor (*SPF*) was estimated by applying the Mansur equation, which calculates *SPF* from the UVB absorbance (290–320 nm) of the extracts. Eight extract concentrations (6.25, 12.5, 25, 50, 100, 200, 250, and 500 µg/mL) were tested. Absorbance measurements were performed in triplicate for each sample. *SPF* was calculated using the formula (Equation (7)):

(7) $$SPF=CF\times \sum_{290}^{320}EE(\lambda )I(\lambda )Abs(\lambda )$$

where *CF* is the correction factor (10), *EE*(*λ*) represents the erythemal effect spectrum, *I*(*λ*) is the solar intensity spectrum, and *Abs*(*λ*) is the measured absorbance of the extract at each wavelength. The *EE* × *I* values are constants as reported by Sayre et al. [24].

### 2.11. Anti-Inflammatory Assay

#### 2.11.1. Erythrocyte Membrane Stabilization Assay

The in vitro anti-inflammatory activity (membrane stabilization) of AM and AC extracts was evaluated using the erythrocyte membrane stabilization method, following the protocol described by Ranasinghe et al. [25]. The Committee of the Institute for the Application of Nuclear Energy (INEP), University of Belgrade, granted ethical approval for this study (Protocol No. 0203-07-013/005/2025), and informed consent was obtained before blood collection. Whole blood was supplied by the Biochemical Laboratory of INEP (Belgrade, Serbia), anticoagulated with sodium oxalate, and stored at 4 °C for up to 24 h. Erythrocytes were separated by centrifugation (770× *g*, 5 min; Thermo Scientific, Waltham, MA, USA), washed three times with sterile isotonic saline, and resuspended in PBS (10 mM, pH 7.4) to obtain a 40% *v*/*v* erythrocyte suspension.

#### 2.11.2. Heat-Induced Hemolysis

For the assay, 1 mL aliquots of isotonic saline containing extract (25, 50, and 100 μg/mL) were mixed with 10 μL of erythrocyte suspension. Controls included isotonic saline without extract (negative control) and isotonic saline containing diclofenac (Galenika, Belgrade, Serbia, 75 μg/mL, positive control). Samples were incubated at 54 °C for 20 min in a water bath (Thermo Scientific™ TSGP10PMO5, Thermo Fisher Scientific, Waltham, MA, USA), while parallel tubes were kept on ice at 4 °C for 20 min. Following incubation, all tubes were centrifuged at 12,300× *g* for 5 min, and the absorbance of the supernatant was measured at 560 nm. The percentage inhibition of erythrocyte hemolysis was calculated using Equation (8) [26]:

(8) $$\%\;of\;the\;lysis\;inhibition\;=100\;\times \;(1-\;\frac{{OD}_{2\;}-\;{OD}_{1\;}}{{OD}_{3\;}-\;{OD}_{1\;}})$$

where *OD*_1_ = absorbance of unheated sample, *OD*_2_ = absorbance of heated sample, and *OD*_3_ = absorbance of heated control. All measurements were performed in triplicate to ensure reproducibility.

#### 2.11.3. Hypotonicity-Induced Hemolysis

The anti-inflammatory potential (membrane stabilization) of extracts under hypotonic stress was evaluated similarly. Briefly, 1 mL of hypotonic saline containing *A. milefolium* or *A. clypeolata* extract (25, 50, and 100 μg/mL) was combined with 10 μL of erythrocyte suspension. A hypotonic solution without extract served as the negative control, while diclofenac (75 μg/mL) in a hypotonic solution was used as a positive control. Samples were incubated at 25 °C for 10 min and then centrifuged at 12,300× *g* for 5 min. The absorbance of the supernatant was measured at 560 nm, and the percentage inhibition of hemolysis was determined using the formula (Equation (9)) [27]:

(9) $$\%\;of\;the\;lysis\;inhibition\;=100\;\times \;(1-\;\frac{{OD}_{2\;}-\;{OD}_{1\;}}{{OD}_{3\;}-\;{OD}_{1\;}})$$

where *OD*_1_ = sample with isotonic solution, *OD*_2_ = sample with hypotonic solution, and *OD*_3_ = control with hypotonic solution. All measurements were performed in triplicate to ensure reproducibility.

Absorbance measurements for the ABTS and DPPH assays, SPF determination, and erythrocyte membrane stabilization assay were carried out using a UV-1800 spectrophotometer (Shimadzu, Kyoto, Japan).

### 2.12. Antimicrobial Assays

The antimicrobial activity of *Achillea* extract samples was examined using the broth microdilution method performed according to the recommendations of the National Committee for Clinical Laboratory Standards (CLSI) [28]. The most common microorganisms that can cause skin infections were used for the analysis, including *Staphylococcus epidermidis* ATCC 12228, *Staphylococcus aureus* ATCC 25923, *Staphylococcus haemolyticus* ATCC 29970, *Streptococcus pyogenes* ATCC 19615, *Pseudomonas aeruginosa* ATCC 27853, *Serratia marcescens* ATCC 14756, and two yeasts, *Candida albicans* ATCC 10231 and *Meyerozyma guillermondii* strain ATCC 6260, as recognized causative agents of skin infections. The antimicrobial activity was determined as MIC (minimum inhibitory concentration) and MBC/MFC (minimum bactericidal concentration/minimum fungicidal concentration).

All bacterial strains were subcultured on the Müller–Hinton agar (MHA), while yeasts were prepared by growing on Sabouraud dextrose agar (SAB). For further preparation, bacterial strains were cultivated overnight in Müller–Hinton broth (MHB), and for fungi, slopes were flooded with 0.85% saline, and conidia were gently probed. The inoculum of the organism was prepared in MHB, and the turbidity was adjusted to approximately 0.5 McFarland turbidity standard to prepare 1 × 10^8^ bacteria/mL. Remaining dilutions in sterile saline were obtained in order to achieve the final concentrations of 1 × 10^6^ CFU/mL for bacteria and 2 × 10^4^ CFU/mL for yeasts.

Briefly, serial dilutions of dry extracts in MHB (for bacteria) or Triptone soya broth (TSB) (for yeasts) were prepared in a microtiter plate with 96 wells. Additionally, 20 μL of microbial suspension and 10 μL of resazurin solution as an indicator of growth were added, reaching a final concentration of 200 μL per well. Microbial strains in an appropriate liquid medium with resazurin solution were used as positive controls, while negative controls contained only the medium with the indicator. The plates were incubated for 24 h at 37 °C for bacteria and for three to seven days at 25 °C for fungi. The MIC was defined as the lowest concentration exhibiting no visible growth, which was indicated by the absence of a color change in the wells. Cell growth was detected in wells with a pink color due to the reduction of purple resazurin to pink resofurin by the activity of living microbial cells. Aliquots from wells showing no visible growth were inoculated into sterile liquid media or onto MHB/SAB agar, depending on the type of microorganism. The MBC was determined after 24 h of incubation at 37 °C, and the MFC was determined after 3 days of incubation at 25 °C, defined as the lowest concentration showing no visible growth. All tests were performed in triplicate.

For antimicrobial activity testing, the positive control for bacteria was gentamicin, and for yeasts, fluconazole was used to confirm assay validity by demonstrating a known inhibitory effect against microbial growth.

### 2.13. Statistical Analysis

In the cell assays, one-way analysis of variance (ANOVA) with Tukey’s post hoc test was used to assess differences between treatments and the control after data were tested for normality. All results are expressed as mean + standard error of the mean (mean ± SEM) or mean ± standard deviation (SD). GraphPad Prism 6.0 (GraphPad Software, Inc., La Jolla, CA, USA) was used for statistical analysis, where *p* < 0.05 was considered significant. Statistical differences among samples in quantitative measurements, spectrophotometric measurements of ABTS and DPPH antioxidant and anti-inflammatory activities, as well as SPF values, were evaluated using one-way ANOVA followed by Duncan’s post hoc test (StatSoft, Tulsa, OK, USA). Differences were considered statistically significant at *p* < 0.05.

## 3. Results and Discussion

### 3.1. UHPLC-MS/MS Identification of Compounds in A. millefolium and A. clypeolata Extracts

The extracts obtained from *A. millefolium* and *A. clypeolata* aerial parts were analyzed by UHPC-MS/MS in a negative ionization mode (Table 1). Thus, out of the 86 compounds, 24 compounds were unambiguously identified by direct comparison with authentic standards, and 62 compounds were tentatively identified based on their chromatographic characteristics (*m*/*z* values, molecular formula, and fragmentation pattern) by comparison with those described in open-access LC-MS libraries MONA, *m*/*z* Cloud, and MassBank [29,30,31], as well as in the literature. The compounds belong to different metabolite classes as described below.

#### 3.1.1. Quinic Acid and Its Acyl Derivatives

Compound **4** had a deprotonated molecular ion [M–H]^−^ at *m*/*z* 191, and its MS/MS gave characteristic quinic acid fragment ions [32]. Further, 5-*O*-caffeoyl (chlorogenic, **15**), 5-*O-p*-coumaroyl (**18**), and 5-*O*-feruloyl (**22**) quinic acids showed deprotonated molecular ions at *m*/*z* 353, 337, and 367, respectively, and the base peak characteristic of C-5 substituted quinic acids at *m*/*z* 191 in their MS/MS. MS/MS of neochlorogenic acid (3-*O*-caffeoylquinic acid, 10) displayed intense peaks at *m*/*z* 179 and 135, in addition to the base peak at *m*/*z* 191 and [M–H]^−^ at *m*/*z* 353. The identification of 1,3-, 3,4-, 3,5-, 1,5-, and 4,5-dicaffeoylquinic acids (20, 45, 49, 50, and 63, respectively) was achieved by direct comparison of their mass spectral data with those of authentic standards. Compounds **40** and **54** had [M–H]^−^ at *m*/*z* 515 and MS/MS fragmentation patterns characteristic of dicaffeoylquinic acids. Additionally, 3,4,5-tricaffeoylquinic acid (69) was deduced from the deprotonated molecular ion [M–H]^−^ at *m*/*z* 677; fragment ions at *m*/*z* 515, 353, and 191, obtained by the loss of three caffeoyl residues; and characteristic fragment ions at *m*/*z* 173, 135, and 179 [33].

Mono- and dicaffeoylquinic acids are common constituents of plants of the Asteraceae family. There are several reports on the presence of these compounds in *A. millefolium* [34,35,36,37,38]. Thus, chlorogenic acid and six dicaffeoylquinic acids with undetermined positions of the ester groups were tentatively identified in the acetone–water extract and ethyl acetate and butanol fractions of *A. millefolium* collected in Switzerland [32]. Similarly, Apel et al. reported the presence of chlorogenic acid and two unspecified dicaffeoylquinic acids in *A. millefolium* from Italy [33]. In another study of *A. millefolium* from Italy, Marengo et al. described the presence of chlorogenic acid and 1,3-, 1,5-, and 3,5- dicaffeoylquinic acids [34]. These compounds, along with 3,4- dicaffeoylquinic acid, have been detected in the methanol extract of *A. millefolium* from Serbia [35]. LC-MS analysis of the methanol extract of *A. millefolium* from Portugal showed the presence of 3-, 4- and 5-caffeoylquinic acids and 3,4-, 4,5-, *cis*- and *trans*-3,5-dicaffeoylquinic acids [36]. The literature survey showed only one article reporting the presence of a chlorogenic acid in *A. clypeolata* [39].

#### 3.1.2. Phenolic Acids and Their Glycosides

Regarding phenolic acids and their glycosides, 4-hydroxybenzoic acid (**14**), 2,5-dihydroxybenzoic acid (**16**), and caffeic acid (**17**) were identified by their deprotonated molecular ions at *m*/*z* 137, 153, and 179, while the characteristic fragment ion corresponded to [M–H–CO_2_]^−^, due to the neutral loss of CO_2_. Compounds **7** and **9** had the same [M–H]^−^ at *m*/*z* 315 and a fragment ion in MS/MS at *m*/*z* 153, due to the elimination of 162 Da, as well as fragments characteristic of dihydroxybenzoic acid. The compounds mentioned were tentatively identified as dihydroxybenzoic acid hexosides. Similarly, compounds **8** and **11** were identified as hydroxy-methoxybenzoic acid *O*-hexoside and syringic acid *O*-hexoside, respectively. Compound **13** was identified as dihydroxybenzoic acid *O*-pentoside, as it had [M–H]^−^ at *m*/*z* 285 and a fragment ion at *m*/*z* 153 as a result of the loss of a pentose (132 Da). Compound **43** was tentatively identified as isoverbascoside.

Previous studies on *Achillea* species demonstrated similar constituents. In methanolic extracts of *A. millefolium* and *A. biebersteinii*, LC–MS/MS analyses have identified a range of phenolic acids, with chlorogenic acid derivatives and hydroxycinnamic acids as predominant components [40]. The investigation of Radušiene et al., related to wild *A. millefolium* from different geographical locations, further supports the dominance of hydroxycinnamic acids such as caffeoylquinic acids, with smaller amounts of free hydroxybenzoic acids like caffeic acid detected in all plant organs [41].

#### 3.1.3. Flavonoids and Their Glycosides

Flavonoids and their glycosides were the major class of compounds identified in *A. millefolium* and *A. clypeolata* extracts. Free aglycones (**64**–**68**, **70**–**75**, **77**–**79**, and **81**–**86**) and flavonoid mono- and di-*O*- and *C*-glycosides were identified according to their mass spectral fragmentation. Compounds **65**, **66**, and **70** had a [M–H]^−^ at *m*/*z* 301, 285, and 269, respectively, and were identified as quercetin, luteolin, and apigenin, respectively, by comparison with authentic standards. Compound **84** had [M–H]^−^ at *m*/*z* 283 and MS/MS fragment ions at *m*/*z* 268 [M–H–CH_3_]^−^, 239 [M–H–CO_2_]^−^, as well as ions at *m*/*z* 151, 117, and 107, due to the Retro-Diels-Alder cleavage and was identified as acacetin (4′-methoxyapigenin) [42]. Compound **64** had [M–H]^−^ at *m*/*z* 287 and MS/MS fragment ions at *m*/*z* 151 (1,3A^−^) and 135 (1,3B^−^), due to the cleavage of the heterocyclic C-ring, as well as at *m*/*z* 107 as a result of a cleavage of the bond linking the B ring. The mentioned compound was identified as the flavanone eriodictyol [43]. Compounds **71**, **72**, and **74** had [M–H]^−^ at *m*/*z* 299 and a base peak at *m*/*z* 284 [M–H–CH_3_]^−^ revealing a methoxy-trihydroxy flavone structure. The presence of the additional intense peaks at *m*/*z* 255 and 227 in MS/MS of the compound **74** allowed its identification as isokaempferide (3-methoxy kaempferol) [44]. The presence of a product ion at *m*/*z* 256 corresponding to the loss of •CH_3_ (15 Da) and neutral CO (28 Da) at different abundances in MS/MS of the compounds **71** and **72** allowed the identification of diosmetin and chrysoeriol, respectively [45]. Compounds **81** and **85** had a [M–H]^−^ at *m*/*z* 313 and fragment ions with different intensities at *m*/*z* 298 [M–H–CH_3_]^−^, 283 [298–CH_3_]^−^, and 255 [283–CO]^−^. They were identified as 5,7′-dihydroxy-6,3′-dimethoxyflavone (pectolinarigenin) and 5,4′-dihydroxy-7,3′-dimethoxyflavone (velutin). Compounds **67** and **73** had a [M–H]^−^ at *m*/*z* 315 and a fragment ion at *m*/*z* 300 [M–H–CH_3_]^−^ with different abundances. The minor fragment ions at *m/z* 151 and 107 in the compound **73** can be assigned to A-ring-containing fragments and allow its identification as isorhamnetin [46], while the compound **67** was identified as 3-methylquercetin [47]. Compounds **75** and **77** displayed [M–H]^−^ at *m*/*z* 329 and fragment ions with different abundances at *m*/*z* 314, 299, and 271 corresponding to the sequential loss of 2 •CH_3_ and neutral CO, revealing a dimethoxy-trihydroxyflavone structure. The mentioned compounds were identified as cirsiliol and jaceosidin. Compounds **82** and **86** displayed [M–H]^−^ at *m*/*z* 343 and fragment ions with different abundances at *m*/*z* 328, 313, and 298, due to the sequential loss of 3 •CH_3_, as well as fragment ions at *m*/*z* 285 [313–CO]^−^ and 270 [298–CO]^−^. They were identified as eupatilin and santin. Compounds **78** and **79** were trimethoxy-trihydroxyflavones, as deduced from their [M–H]^−^ at *m*/*z* 359 and the prominent fragment ions, due to the sequential loss of 3 •CH_3_. The compounds were identified as jaceidin and centaureidin. Compound **83** displayed [M–H]^−^ at *m*/*z* 373 and fragment ions in MS/MS corresponding to a tetramethoxyflavone of the quercetagetin type and was identified as chrysosplenetin [48].

Quercetin, luteolin, kaempferol, apigenin, patuletin, isorhamnetin, chrysoeriol, diosmetin, petunidin, and hispidulin mono- and di-*O*-glycosides (23, 26, 27, 29–31, 33–39, 41, 42, 44, 46–48, 51–53, 55, and 57–62) were identified by the characteristic fragment ions in their MS/MS, due to the elimination of rutinosyl (308 Da), hexosyl (glucosyl or galactosyl) (162 Da), glucuronyl (176 Da), xylosyl or arabinosyl (132 Da), etc., moieties from the corresponding deprotonated molecular ions. In the case of luteolin 7,3′-di-*O*-glucoside (**23**), the presence of another intense fragment ion at *m*/*z* 447 in the MS/MS spectrum of the compound **23** was consistent with a compound with sugar moieties attached at different carbon atoms [49].

Compound **24** had a [M–H]^−^ at *m*/*z* 447 and a fragment ion at *m*/*z* 285 [M–H–162]^−^, due to the loss of a hexoside moiety, and was tentatively characterized as luteolin hexoside. Further, the diagnostic ion corresponding to the base peak for C-6-hexosides at *m*/*z* 327 [M–H–120]^−^ was of higher abundance than that of [M–H–90]^−^ at *m*/*z* 357 and allowed identification of compound **24** as homoorientin (luteolin 6-*C*-glucoside) [50]. Compound **32** had [M–H]^−^ at *m*/*z* 447 and characteristic fragment ions in the MS/MS spectrum of apigenin C-glucoside. The position of the glucoside moiety at C-8 was deduced from differences in the relative abundance of fragment ions at *m*/*z* 341 and 283 [51]. Thus, compound **32** was identified as vitexin. Compounds **21** and **25** were identified as apigenin 6,8-di-C-glucoside and schaftoside (apigenin 6-glucoside-8-arabinoside) from the corresponding deprotonated molecular ions at *m*/*z* 593 and 563, and the characteristic fragmentation pattern [52].

The studied *A. millefolium* and *A. clypeolata* extracts showed a similar qualitative profile regarding flavonoids and their glycosides. Thus, out of 53 detected flavonoids, 29 compounds were common to both species, and the additional 14 and 10 compounds were detected in *A. millefolium* and *A. clypeolata*, respectively. As can be seen from Table 1, *A. clypeolata* was richer in flavonoid aglycones than *A. millefolium*. The flavonoid profile of *A. millefolium* was consistent with literature data [34,35,36,37,38,53]. The literature data for the flavonoid content of *A. clypeolata* is scarce. Centaureidin has been isolated from *A. clypeolata* [11], while several flavonoids (luteolin 7-glucoside, hesperidin, hyperoside, apigenin 7-glucoside, eriodictyol, quercetin, luteolin, kaempferol, and apigenin) have been detected by LC/MS in the methanol extract of *A. clypeolata* [39].

#### 3.1.4. Other Compounds

Two coumarin derivatives (esculin (**12**) and isofraxidin (**28**)), malic acid (**6**), azelaic acid (**56**), carbohydrates (**1**–**3** and **5**), and fatty acids (**19**, **76**, and **80**) were also tentatively identified by comparison of their mass spectral data with those in the open-access libraries. Coumarins have been previously reported as constituents of *Achillea* species [54,55].

### 3.2. HPLC Quantification of Compounds

#### 3.2.1. HPLC Analysis of Chlorogenic and Dicaffeoylquinic Acids

The most intense peaks in the TIC chromatograms of *A. millefolium* and *A. clypeolata* were those corresponding to 5-caffeoylquinic (5-CQA) and 3,5-, 1,5-, 4,5-, and 3,4-dicaffeoylquinic (DCQA) acids, and their amounts were further determined using the HPLC-PDA method at 320 nm. The obtained results (Table 2) showed that *A. millefolium* was richer in caffeoylquinic acids (63.57 mg/g dry extract) in comparison with *A. clypeolata* (39.59 mg/g dry extract). Further, 3,5-DCQA was the major compound in both extracts, followed by 5-CQA, 4,5-DCQA, 1,5-DCQA, and 3,4-DCQA. Our results regarding *A. millefolium* extract were in accordance with those published recently by Cvetković et al. [37]. The authors also reported 3,5-DCQA (7.839%) as a major component in the methanol extract, followed by 1,5-DCQA (3.497%), 5-CQA (2.216%), and 3,4-DCQA (0.999%). Similarly, 3,5-DCQA and 5-CQA were the major compounds in methanol extracts from different plant parts (inflorescences, leaves, and stems) of *A. millefolium* from Turkey and Lithuania [41]. However, significant variation in the amount of caffeoylquinic acids was observed, both between different plant parts and geographical locations. Caffeoylquinic acids were predominant in the leaves of plants. Additionally, 1,5-DCQA was one of the main compounds in all plant parts of the Lithuanian sample, while in the Turkish population, this compound was detected in small amounts only in leaves and stems [41]. In another study of *A. millefolium* extracts, the amounts of 1,5-DCQA and 5-CQA varied from 16.0 µg/mg to 68.4 µg/mg of dry extract and from 9.0 µg/mg to 57.6 µg/mg of dry extract [36]. To the best of our knowledge, this is the first report on the quantitative determination of caffeoylquinic acids in *A. clypeolata*.

#### 3.2.2. HPLC Analysis of Flavonoids

The genus *Achillea* is widely recognized for its high flavonoid content [4]. In the present study, *A. clypeolata* and *A. millefolium* were compared for the first time in terms of their flavonoid profiles. The comparative evaluation revealed pronounced qualitative and quantitative differences between the two species (Table 3). *Achillea millefolium* exhibited higher concentrations of rutin, hyperoside, cosmosiin, cynaroside, and quercetin, reflecting a greater abundance of luteolin-, quercetin-, and apigenin-derived flavonoids. Consistent with our findings, Benetis et al. reported that flavonoid distribution patterns in yarrow flower samples were dominated by apigenin 7-*O*-glucoside (3.334–7.546 mg/g) and luteolin 7-*O*-glucoside (2.107–5.885 mg/g) [56]. The rutin content quantified in our sample (~2.44 mg/g) was slightly lower than the value reported by Trumbeckaite et al. (3.2 mg/g), suggesting moderate variability that may arise from differences in plant origin, extraction protocols, or analytical methodologies [57].

In contrast, *A. clypeolata* was distinguished by elevated levels of narcissoside and nicotiflorine, corresponding to isorhamnetin and kaempferol derivatives, respectively, and was the only species in which isorhamnetin was detected. To the best of our knowledge, this study represents the first report on the quantitative determination of individual flavonoid compounds in *A. clypeolata*.

### 3.3. FT-IR Spectra of Achillea milefolium and Achillea clypeolata Extracts

**Figure 1:**
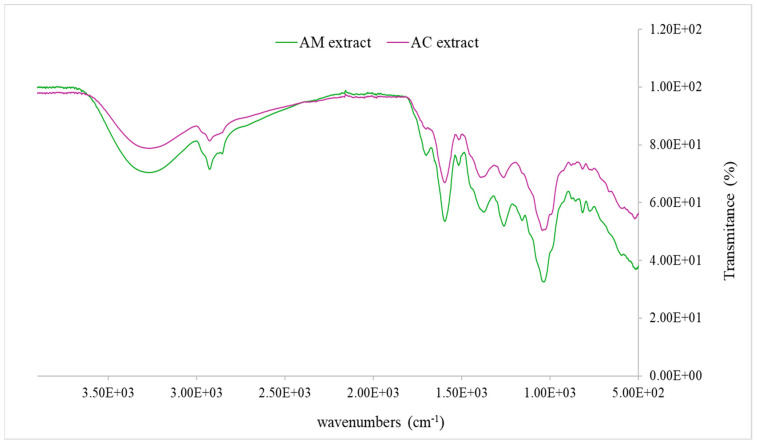
Fourier Transform Infrared (FT −IR) spectra of *Achillea millefolium* (AM) and *Achillea clypeolata* (AC) extracts.

The FT-IR spectra of *A. millefolium* and *A. clypeolata* extracts are shown in Figure 1. The FT-IR spectra of both extracts display characteristic functional group signatures indicating phenolic, aromatic, and possibly sesquiterpene constituents.

In *A. millefolium*, major peaks are seen at strong C–O stretching in the ~1000–1250 cm^−1^ range, ~1600 cm^−1^ (aromatic C=C), alongside aliphatic C-H bands (~2920–2850 cm^−1^), and a broad O–H stretch (~3300–3400 cm^−1^) indicating abundant hydroxyl groups. These features are consistent with high content of flavonoids, phenolic acids, and glycosides. The band at ~1515 cm^−1^ corresponds to aromatic C=C stretching vibrations, characteristic of the flavonoid backbone, thereby confirming the presence of flavonoid constituents, such as apigenin and luteolin derivatives. The band around 1380 cm^−1^ is attributable to O–H in-plane bending of phenolic groups and aromatic C–H deformations, with potential contributions from symmetric COO^−^ stretching of organic acids. The band detected at ~815 cm^−1^ can be assigned to out-of-plane C–H bending of aromatic rings, consistent with *p*-substituted phenolic structures characteristic of flavonoid compounds in *Achillea* extracts. *A. millefoilum* extract exhibited more intense bands at ~3300–3400 cm^−1^ and ~2925 cm^−1^, which suggests a higher proportion of free phenolic hydroxyl groups compared to *A. clypeolata* extract. The intensity of peaks at ~1000–1250 cm^−1^ and ~1600 cm^−1^ was also higher in the case of *A. millefoilum*. For *A. clypeolata*, its spectra show strong aromatic C=C bands and phenolic C–O and O–H signals, suggesting the presence of phenolic and sesquiterpene compounds. The FT-IR spectrum of the AC extract displayed characteristic absorption bands corresponding to the major functional groups of phenolic and flavonoid constituents [10,58]. A broad, intense band in the region 3400–3200 cm^−1^ was assigned to O–H stretching vibrations of hydroxyl groups, typical of polyphenols, i.e., flavonoids. The absorption around 2920–2850 cm^−1^ corresponded to C–H stretching vibrations of aliphatic –CH_2_ and –CH_3_ groups. A strong band near 1720–1700 cm^−1^ was attributed to C=O stretching of carbonyl groups, indicating the presence of phenolic acids and flavonoid glycosides. The band at 1630–1600 cm^−1^ was assigned to aromatic C=C stretching vibrations of flavonoid and phenolic backbones. Additional signals in the region 1510–1450 cm^−1^ reflected C=C skeletal vibrations of aromatic rings, while bands at 1370–1320 cm^−1^ suggested C–H bending and possible phenolic O–H deformation. Strong absorptions in the 1260–1020 cm^−1^ range corresponded to C–O stretching of alcohols, ethers, and glycosidic linkages. Finally, signals below 900 cm^−1^ indicated aromatic C–H out-of-plane bending, consistent with substituted phenolic structures. Differences in glycosylation, degree of esterification, and bound phenolic content will further modulate the profiles [59]. Comparing the two samples, the AM extract showed greater phenolic-related spectral signatures (also shown in the HPLC analysis), which has been associated in many studies with higher antioxidant, antimicrobial, or free radical scavenging activity. In contrast, the AC sample likely has complementary bioactivity driven in part by its terpenoid content, which, though less polar, may contribute more to antimicrobial or lipophilic interactions [4,60,61]. FT-IR helps illustrate these compositional distinctions even before chromatographic or mass spectrometric profiling, supporting decisions on extraction solvent choice, fractionation, and bioactivity prediction.

### 3.4. Enzyme Inhibiting Potential

The anti-aging potential of *A. millefolium* and *A. clypeolata* leaf ethanolic extracts was evaluated using four in vitro enzymatic assays using collagenase, elastase, hyaluronidase, and tyrosinase. The mentioned enzymes are connected with skin aging, and the inhibition or modulation of their activities could preserve skin elasticity, firmness, moisture retention, integrity, and overall health. Several previous studies showed anti-enzyme activities of herbal extracts and isolated natural compounds.

Elastin, which is an elastic fiber present in tissues, provides tissue elasticity and contributes to this youthful appearance. Its production decreases during maturity, while damage and degradation can be induced by a variety of factors, such as chronic sun exposure [13,62,63]. Collagen, which is mainly present in connective tissues, including skin, joints, and bones, accounts for 70% to 80% of dry skin weight. It further contributes to maintaining cutaneous strength, elasticity, and thickness by preserving normal epidermal–dermal interactions [64]. Among the essential constituents of the skin’s extracellular matrix, hyaluronic acid plays a key role in binding water and maintaining optimal hydration, moisture, smoothness, and lubrication. Finally, tyrosinase is the enzyme responsible for mediating melanin formation in the skin, and suppression of its activity may reduce hyperpigmentation [65].

**Figure 2:**
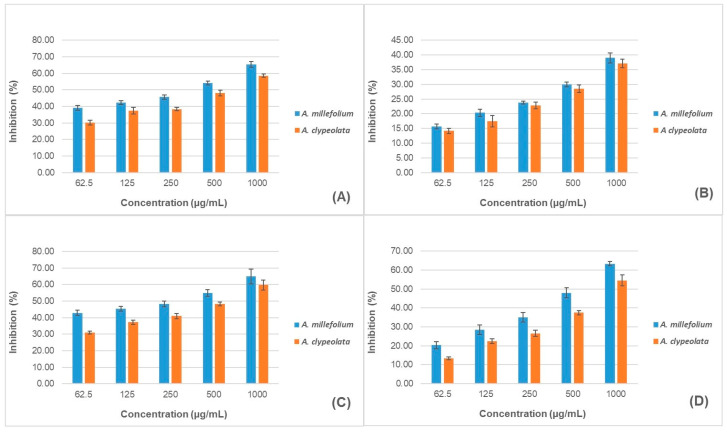
Enzyme inhibiting activity of *Achillea millefolium* and *A. clypeolata* leaf ethanolic extracts at different concentrations against (**A**) collagenase, (**B**) elastase, (**C**) hyaluronidase, and (**D**) tyrosinase.

In our study, the IC_50_ values of the tested extracts (Table 4) were calculated based on inhibition values at the tested concentration range and compared to those of the positive controls. The extracts exhibited dose-dependent inhibition of all enzymes within the concentration range of 62.5 µg/mL to 1000 µg/mL (Figure 2). Both extracts inhibited all four enzymes, but their IC_50_ values were higher than those of the positive controls. *A. millefolium* extract had lower IC_50_ values in each assay (Table 4), indicating stronger inhibition across the tested concentration range compared to *A. clypeolata* extract. The extracts exhibited the highest inhibitory activity against hyaluronidase, whereas their activity against elastase was the lowest (Figure 2).

In vitro anti-tyrosinase activity of water, methanol, and ethyl acetate extracts of *A. millefolium* has been reported by Zengin et al., with IC_50_ values of 15.23, 23.26, and 31.57 mg of kojic acid equivalents (KAE) per g, respectively [66]. Moreover, tyrosinase inhibitory activity was also reported for hydroglycolic extracts of *A. millefolium* [67], which were shown to inhibit both monophenolase and diphenolase activities of this enzyme. Recently, Michalak et al. reported a concentration-dependent ability of the hydroalcoholic extract of *A. millefolium* L. var. *paprika* to inhibit collagenase and elastase activities [68]. At a concentration of 5.0%, inhibition of collagenase was stronger (43.8%) compared to that of elastase inhibition (19.2%). Moreover, inhibition of tyrosinase of 15.5% and 23.4% was reported for concentrations of 1% and 5%, respectively.

In contrast to the extensively studied *A. millefolium*, data on the anti-enzymatic activity of *A. clypeolata* remain limited. Barak et al. reported time-dependent anti-collagenase, anti-hyaluronidase, and anti-elastase activity of a 0.5 mg/mL methanolic extract of *A. clypeolata* measured at five different time points (10, 20, 40, 60, and 80 min), but studies examining the anti-enzyme activity of this species are lacking [39].

The observed differences in the intensity of inhibition among specific enzymes between species may be attributed to variations in phytochemical composition. One of the dominant compounds in both *A. millefolium* and *A. clypeolata* extracts was chlorogenic acid, for which anti-elastase [69], anti-collagenase [70], anti-tyrosinase [71], as well as anti-hyaluronidase activity were reported [72]. The better anti-enzyme activity of AM may be related to the fact that the extract of this species was richer in caffeoylquinic acids.

Moreover, flavonoids and their glycosides were the major class of compounds identified in both AM and AC extracts, and out of 53 detected flavonoids, 29 compounds were common to both extracts. Flavonoids are also known as anti-enzyme agents, whose abundance in AM and AC extracts could also contribute to their activity. Recently, Okselni et al. systematized studies dealing with the activity of quercetin on skin conditions and diseases, pointing out its antioxidant, anti-inflammatory, anti-aging, and wound-healing potential. Moreover, quercetin is a potent inhibitor of tyrosinase, leading to reduction of melanin production and hyperpigmentation [73].

Acikara et al. showed that two of the most abundant compounds in *A. millefolium*, cosmosiin (apigenin 7-*O*-glucoside) and cynaroside (luteolin 7-*O*-glucoside), have potent enzyme-inhibitory activities against elastase and collagenase, which probably contribute to the activities of this species [74]. Also, significant anti-enzyme activity has been reported for two other compounds that are represented to a great extent in *A. millefolium*, rutin and hyperoside [69,75].

### 3.5. Cytotoxicity of Achillea milefolium and Achillea clypeolata Extracts

**Figure 3:**
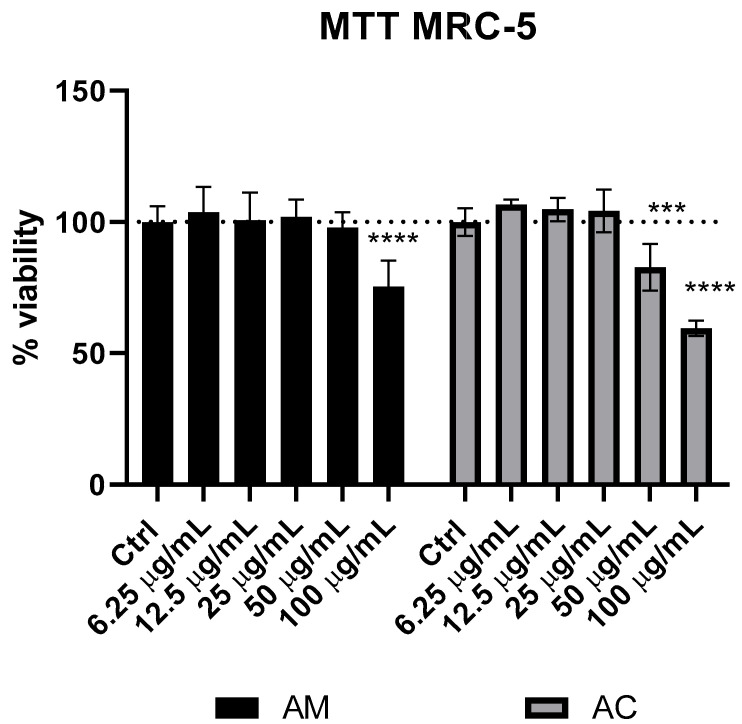
Cytotoxicity of extracts of *Achillea millefolium* (AM) and *Achillea clypeolata* (AC) in a range of concentrations (6.25, 12.5, 25, 50, and 100 μg/mL), determined by the MTT assay in the human fetal lung fibroblasts (MRC-5). Data are expressed as mean + standard error of the mean (*** *p* < 0.001 and **** *p* < 0.0001 versus control—Ctrl) by one-way analysis of variance (ANOVA) with Tukey’s multiple comparison post hoc test, *n* = 6; MTT, thiazolyl blue tetrazolium bromide. The dashed line indicates the control value reference.

The cytotoxic potential of AM and AC extracts was examined in MRC-5 fibroblasts through the MTT assay. This evaluation helped establish the safety margins of the extracts and define the concentration ranges considered non-toxic, providing a basis for further assessment in antioxidant, membrane-stabilizing, and photoprotective applications. The data are graphically presented in Figure 3.

The cytotoxic effects of AM and AC were assessed via the MTT assay in MRC-5 fibroblasts following 24 h of treatment with increasing concentrations (6.25–100 µg/mL, Figure 3). Both extracts exhibited a concentration-dependent reduction in cell viability and a similar cytotoxicity profile. Statistically significant decreases were observed from 50 µg/mL onwards in cells treated with the extracts, with the highest concentration (100 µg/mL) resulting in pronounced cytotoxicity in both AC and AM extracts. The AC extract was more cytotoxic at 100 µg/mL than the AM extract (41% viability reduction versus 25% viability reduction). Also, at a 50 µg/mL concentration, AC induced a decrease in the number of viable cells, while AM did not display a cytotoxic effect at the same concentration, indicating AC has a more powerful effect on MRC-5 cell viability. These findings are consistent with previous reports indicating that *Achillea* species exhibit differential cytotoxic potential depending on their phytochemical composition [76,77,78], particularly the content of flavonoids, sesquiterpene lactones, and phenolic acids, which are known to interfere with cellular redox balance and mitochondrial function [79,80]. Although AM contained higher levels of flavonoids and caffeoylquinic acid derivatives according to HPLC analysis, the stronger cytotoxicity observed for AC may be attributed to qualitative differences in its phytochemical profile, particularly the presence of bioactive terpenoids and aglycone-type phenolic compounds. Specifically, despite the higher abundance of flavonoids and caffeoylquinic acid derivatives in AM (shown in Section 3.2 and Section 3.3), these compounds are predominantly present as glycosides, which may exhibit limited cytotoxic activity. In contrast, the enhanced cytotoxicity of AC is likely associated with its higher content of bioactive terpenoids and lipophilic constituents, which are known for their strong antiproliferative and pro-apoptotic properties in mammalian cell systems [81,82]. The glycosylation of flavonoids increases their polarity and reduces their passive membrane permeability, resulting in lower cellular uptake compared to their corresponding aglycones. This effect has been demonstrated in Caco-2 permeability models, where flavonoid glycosides exhibited significantly reduced transepithelial transport and cellular uptake relative to their aglycone counterparts [83]. Chlorogenic acid and dicaffeoylquinic acid derivatives are well known for their strong antioxidant and cytoprotective properties and have been reported to attenuate oxidative stress-induced cell damage, which may counteract or mask cytotoxic effects in cell-based assays [84]. Consequently, their higher abundance in AM extracts may contribute to the overall lower cytotoxic response observed.

### 3.6. Antioxidant Effects of Achillea milefolium and Achillea clypeolata Extracts

**Figure 4:**
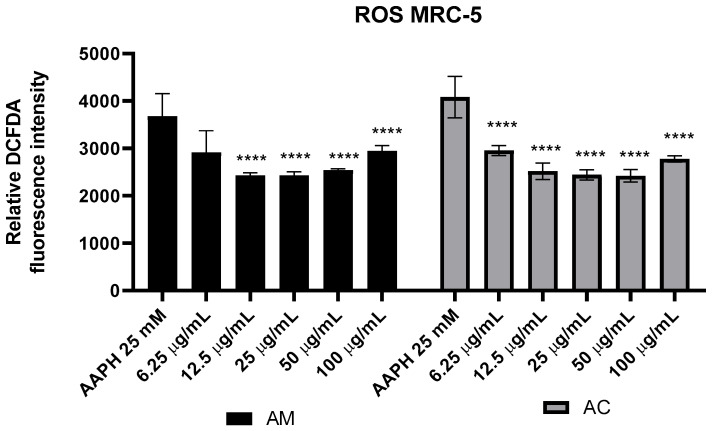
The effect of increasing concentrations of *Achillea millefolium* (AM) and *Achillea clypeolata* (AC) (at concentrations of 6.25, 12.5, 25, 50, and 100 μg/mL) on intracellular ROS (reactive oxygen species) scavenging capacity under conditions of AAPH-induced oxidative stress in MRC-5 cells, measured by DCFDA fluorescence. The data is expressed as mean + standard error of the mean (**** *p* < 0.0001 versus AAPH) by one-way analysis of variance (ANOVA) with Tukey’s multiple comparison post hoc test, *n* = 6; AAPH, 2,2′-azobis(2-methylpropionamidine) dihydrochloride.

The antioxidant potential of AM and AC extracts was assessed using both cell-based and spectrophotometric approaches. Intracellular ROS production in MRC-5 cells exposed to AAPH was measured after treatment with non-cytotoxic concentrations of the extracts (Figure 4). In parallel, their free radical scavenging capacity was determined through ABTS and DPPH assays (Table 5). Together, these methods allowed evaluation of the extracts’ ability to attenuate oxidative stress and neutralize reactive species, highlighting their protective effects at concentrations deemed non-toxic.

The results show that AAPH treatment significantly elevated ROS levels in MRC-5 cells, confirming its oxidative stress-inducing effect. Both *Achillea* extracts, AC and AM, showed dose-dependent antioxidant activity, with higher concentrations leading to greater reductions in ROS levels up to a concentration of 50 µg/mL, highlighting their ability to scavenge free radicals and restore cellular redox balance. In contrast, at 100 µg/mL, both extracts showed weaker antioxidant activity than at lower concentrations, which may be attributed to pro-oxidant effects of polyphenols at higher concentrations, a phenomenon previously described for plant-derived antioxidants [85,86]. The most pronounced decreases in ROS were observed at 12.5, 25, and 50 µg/mL, for both examined extracts, suggesting these doses strike an optimal balance between radical scavenging and avoiding pro-oxidant effects. Specifically, caffeoylquinic acid derivatives have been identified as key antioxidants in *Achillea* species [4,55]. Although AM contained higher levels of flavonoids and caffeoylquinic acid derivatives, its antioxidant activity was comparable to that of AC, except at the lowest tested concentration (6.25 µg/mL), where AC exhibited greater potency. This may be attributed to qualitative differences in phytochemical composition, including the presence of rapidly reacting antioxidant constituents in AC and the predominance of glycosylated flavonoids in AM, which may require higher concentrations to exert their full antioxidant potential in the cell systems [87]. These findings suggest that the two extracts have comparable antioxidant potential, effectively mitigating AAPH-induced oxidative stress in a concentration-dependent manner, with the most effective concentrations being up to 50 µg/mL.

To compare the radical scavenging activity of the investigated *Achillea* species extracts, in vitro spectrophotometric assays were conducted, and the results are summarized in Table 5.

The ABTS radical scavenging assay revealed that AM and AC extracts exhibit notable antioxidant activities, with IC_50ABTS_ values of 22.5 µg/mL and 18.3 µg/mL, respectively. These values suggest that both species possess significant radical scavenging potential. However, direct comparisons with existing literature are limited due to variations in extraction methods, solvent systems, and assay conditions. For instance, a study by Salomon et al. reported an IC_50_ value of 17.0 µg/mL for AM, which is slightly higher than our findings [34]. This discrepancy could be attributed to differences in extraction techniques and solvent polarity. Additionally, Dekanski et al. determined an IC_50_ value of 685.6 µg/mL for AC in a DPPH assay, indicating a lower antioxidant activity compared to AM [88]. The DPPH radical scavenging assay revealed that both AM and AC extracts possess notable antioxidant activity, with IC_50_ values of 381.93 µg/mL and 303.68 µg/mL, respectively (Table 4). The lower IC_50_ of AC indicates a stronger free radical neutralizing capacity compared to AM under the same experimental conditions. This difference may reflect variations in the phytochemical composition between the two species, particularly the presence of more rapidly reacting antioxidant constituents such as aglycone flavonoids and bioactive terpenoids, whereas the higher content of glycosylated flavonoids and caffeoylquinic acid derivatives in AM may require higher concentrations to exert comparable radical neutralization [34,87,89,90]. These findings align with previous studies demonstrating that *Achillea* species exhibit dose-dependent antioxidant properties, mediated primarily by phenolic acids, flavonoids, and terpenoid constituents, which effectively quench DPPH radicals and reduce oxidative stress in vitro [58,91]. This variation underscores the importance of standardizing experimental conditions when comparing antioxidant potentials across studies. The observed antioxidant activities of AM and AC are likely influenced by their phytochemical compositions. AM is known to contain high levels of flavonoids and phenolic acids, which are potent antioxidants (also shown in Section 3.2 and Section 3.3 by HPLC analysis). AC, on the other hand, is rich in sesquiterpene lactones, contributing to its bioactivity. The presence of these bioactive compounds in both species supports their traditional use in herbal medicine for treating oxidative stress-related conditions. Both AM and AC extracts demonstrate significant radical scavenging activities, with AC exhibiting a marginally stronger effect than AM. The antioxidant potentials of these species are attributed to their distinct phytochemical profiles, including flavonoids, phenolic acids, and sesquiterpene lactones. Further studies employing standardized extraction and assay methods are necessary to validate these findings and facilitate comparisons with other plant species.

### 3.7. In Vitro Sun Protection Factor of Achillea millefolium and Achillea clypeolata Extracts

**Figure 5:**
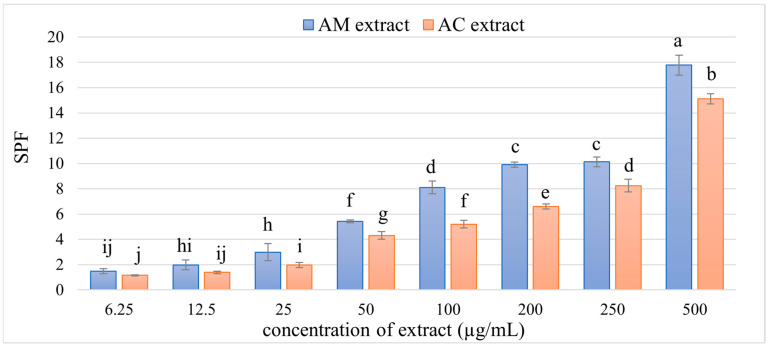
In vitro sun protection factor (SPF) of *Achillea millefolium* (AM) and *Achillea clypeolata* extracts (AC). Different letters indicate statistically significant differences between various extracts and concentrations (*p* < 0.05, *n* = 3), determined by one-way ANOVA followed by Duncan’s post hoc test.

The UV-absorbing properties of AM and AC extracts were evaluated by recording their absorbance across the UV spectrum. All samples exhibited notable absorption throughout the UV-A, UV-B, and UV-C regions. The calculated SPF values for extract concentrations of 6.25, 12.5, 25, 50, 100, 200, 250, and 500 μg/mL are presented in Figure 5.

The SPF values obtained for AM extract indicate a clear dose-dependent photoprotective effect. At the lowest concentration of 6.25 µg/mL, the extract exhibited an SPF of 1.47, which gradually increased to 17.77 at 500 µg/mL, demonstrating that higher concentrations of the extract provide significantly greater UV absorption (Figure 5). These findings are in line with previous reports on *A. millefolium*, although differences in SPF values are observed due to variations in extraction methods, solvents, and plant material sources. For instance, Berbatovci-Ukimeraj et al. reported an SPF of 35.5 for an ethyl acetate extract obtained via Soxhlet extraction, which is higher than our values, likely due to the more efficient extraction of photoprotective compounds [92]. Similarly, Gaweł-Bęben et al. observed SPF values of 14.04 for water:polyethylene glycol extracts, highlighting that solvent polarity and extraction ratios significantly influence the chemical composition and subsequent photoprotective efficacy of the extracts [67]. The photoprotective activity of *A. millefolium* is largely attributed to its rich content of flavonoids such as luteolin and apigenin, phenolic acids, and essential oil components like proazulene, all of which exhibit UV-absorbing and antioxidant properties. These compounds likely act synergistically to absorb UV radiation and mitigate oxidative stress in skin cells. Methodological considerations, including the spectrophotometric assessment of SPF and the choice of solvent and extraction method, are important factors that contribute to the observed variability in literature reports. The slightly different SPF values observed in our study may be attributed to variations in total phenolic and flavonoid content or differences in the extract preparation and solvent polarity. This is consistent with reports indicating a positive correlation between total phenolic content and SPF values in plant extracts [93].

The in vitro SPF evaluation of AC extract also demonstrated a clear dose-dependent increase in photoprotective activity. At the lowest tested concentration (6.25 µg/mL), the SPF was 1.15, while at the highest concentration (500 µg/mL), the SPF reached 15.1, indicating that the extract exhibits moderate UV-filtering properties at higher concentrations (Figure 5). The gradual increase in SPF with increasing concentration suggests that the bioactive compounds in the extract, particularly phenolic acids and flavonoids, contribute significantly to the absorption of UV radiation. Flavonoids, such as apigenin, luteolin, and quercetin derivatives, are known to absorb in the UVA and UVB regions, acting as natural UV filters [94,95,96]. Additionally, the presence of polyphenols may enhance the photostability of the extract by neutralizing free radicals generated under UV exposure, further contributing to photoprotection [67,97].

The data suggest that AM and AC could be potential natural photoprotective agents, and their efficacy could be further enhanced through optimization of extraction methods and formulation strategies [68]. The SPF values of AM extract were consistently higher than those of AC extract across all tested concentrations, indicating a stronger photoprotective effect. This suggests that AM extract contains a higher concentration or more effective composition of UV-absorbing compounds, such as flavonoids and phenolic acids, compared to AC extract (also shown in the HPLC analysis in Section 3.2 and Section 3.3). The moderate SPF at 200–250 µg/mL indicates suitability for inclusion in topical formulations, either as a standalone ingredient or in combination with other natural or synthetic UV filters to achieve a higher SPF. Further studies, including in vivo SPF testing, photostability assessment, and formulation studies, are necessary to confirm the efficacy of these extracts in cosmetic or dermatological applications.

### 3.8. In Vitro Anti-Inflammatory (Membrane Stabilization) Activity of Achillea millefolium and Achillea clypeolata Extracts

**Figure 6:**
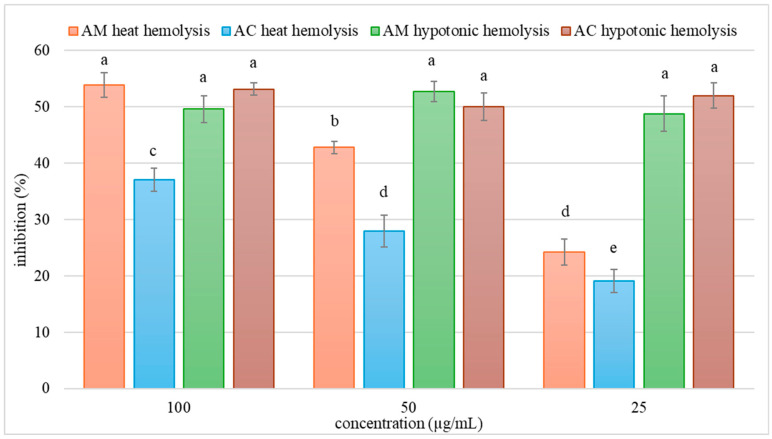
Effects of *Achillea millefolium* (AM) and *Achillea clypeolata* (AC) extracts on erythrocyte membrane stability under heat- and hypotonicity-induced hemolysis conditions at different concentrations; different letters showed statistically significant differences (*p* < 0.05, *n* = 3, one-way analysis of variance followed by Duncan’s post hoc test).

The anti-inflammatory (membrane stabilization) potential of AM and AC extracts was evaluated using the erythrocyte membrane stabilization assay, employing both heat- and hypotonicity-induced hemolysis models. Results for the different extract concentrations are summarized in Figure 6.

The present study demonstrates that AM and AC extracts inhibit red blood cell (RBC) hemolysis under both heat-induced and hypotonic stress. Notably, AM exhibits significantly greater inhibition under heat (oxidative) stress, while under hypotonic (osmotic) stress, both extracts perform comparably. These findings suggest both membrane-stabilizing and antioxidant actions, with species-specific differences. The inhibition of heat-induced hemolysis by plant extracts may be attributed to their antioxidant potential. Heat stress induces lipid peroxidation and protein oxidation in erythrocyte membranes, leading to cell lysis [98]. Previous studies support this observation. Specifically, Asgari et al. demonstrated that *A. millefolium* extract significantly reduces oxidative hemolysis and preserves sulfhydryl groups in RBCs [99]. Similarly, Konyalioglu et al. reported high antioxidant activity in *Achillea* infusions, correlating with protection against oxidative damage in human erythrocytes and leukocytes [100]. Specifically, the study assessed the ability of various *Achillea* species infusions to counter H_2_O_2_-induced oxidative damage in blood cells, and AM was among the better performers in preserving antioxidant enzyme activity and reducing lipid peroxidation [100]. This report aligns with our result that AM more strongly protects against heat-induced (presumably oxidative) hemolysis compared to AC. The enhanced RBC membrane stabilization observed for AM relative to AC can also be related to its greater abundance of flavonoids and caffeoylquinic acid derivatives (Section 3.2 and Section 3.3, HPLC analysis data), which can protect erythrocyte membranes from oxidative damage and improve cellular resilience. On the other hand, detailed data on AC effects on the RBCs, i.e., hemolytic or antihemolytic activity, are lacking.

Hypotonic-induced lysis primarily results from osmotic swelling and rupture of the RBC membrane. The effect of *Achillea* extracts dropped at lower concentrations, particularly for heat stress, but remained relatively high for hypotonic lysis even at lower doses (Figure 6). This suggests different thresholds/modes of action: antioxidants may require higher doses to quench enough ROS, whereas membrane-stabilizing interactions may be effective even at moderate concentrations. Additionally, membrane stabilization under hypotonic or osmotic stress tends to correlate with the presence of phenolics, such as flavonoids, and other membrane-active compounds. Specifically, protection in this context likely involves phytochemicals that stabilize the lipid bilayer or modulate membrane fluidity [101]. Both tested *Achillea* extracts showed strong and statistically similar inhibition of hypotonic hemolysis, suggesting the presence of membrane-stabilizing constituents in both species, which echoes their strong membrane-stabilizing capacity. The observed activity may relate to the presence of flavonoids, sesquiterpenes, phytosterols, and oxygenated monoterpenes in *Achillea* species, known to reinforce membrane integrity [4]. Such compounds are known to scavenge ROS, protect lipids from peroxidation, and interact with cell membranes to stabilize them. Some terpenoids may integrate into lipid membranes, affecting fluidity, and may have mild antioxidant activity. Furthermore, phytosterols (β-sitosterol and stigmasterol) may also affect the membrane microenvironment, i.e., membrane stiffness/fluidity and resistance to osmotic stress. The phytochemical composition of *Achillea* species is known to vary, but *A. millefolium* has been reported to contain flavonoids (e.g., luteolin, apigenin), caffeoylquinic acids, and essential oil with strong antioxidant properties [34,101,102]. *A. clypeolata*, though less studied, contains sesquiterpenes, diterpenes, and flavonoids [10], which may contribute to its membrane-protective effects under osmotic stress. Our findings are consistent with prior studies indicating that the antihemolytic activity of plant extracts correlates with their phenolic and flavonoid contents [34,103,104]. While both AM and AC possess polyphenol constituents, the stronger performance of AM under oxidative conditions suggests a higher concentration (also shown via HPLC analysis of the extracts, Section 3.2 and Section 3.3) or more potent composition of these bioactives. Thus, the combined antioxidant and membrane-modulating phytochemistry likely explains the dual protective effect and the differences between species. In conclusion, the results support the ethnopharmacological use of *Achillea* species, particularly *A. millefolium*, as antioxidant and membrane-stabilizing agents.

### 3.9. Antimicrobial Activity

In our study, the antimicrobial activity of *A. millefolium* and *A. clypeolata* extracts was tested against selected Gram-positive and Gram-negative bacteria and yeasts, which are known as potential skin pathogens. *Staphylococcus epidermidis* and *S. haemolyticus* are normal skin residents that contribute to homeostasis but can act as opportunistic pathogens through biofilm formation and antibiotic resistance, particularly in compromised skin or immunosuppressed individuals. *Staphylococcus aureus*, including resistant strains, is a leading cause of skin and soft tissue infections, responsible for conditions ranging from superficial lesions to severe invasive disease. *Streptococcus haemolyticus* is an important contributor to common inflammatory skin infections, such as impetigo and cellulitis, due to its ability to alter skin layers and immune defenses [105,106,107]. Gram-negative bacteria, such as *P. aeruginosa*, commonly infect burns, chronic wounds, and moist skin sites, where toxins and biofilms impair healing and promote severe infections, while *S. marcescens*, although it rarely infects skin, can cause severe ulcers or necrotizing infections in immunosuppressed individuals. The yeasts *C. albicans* and *M. guilliermondii* were chosen to represent fungal components of the skin microbiome that can overgrow under dysbiotic or immunocompromised conditions and contribute to chronic or inflammatory skin disorders [105,106,107].

Our results showed that both tested plant extracts possess antimicrobial potential, with activity varying according to the tested microorganism (Table 6). Overall, Gram-positive bacteria were more susceptible to both *Achillea* extracts than Gram-negative bacteria. Among the tested strains, *S. epidermidis* showed the highest sensitivity, with very low MIC values (0.395–0.397 mg/mL) and relatively low MBC values (~1.58 mg/mL) for both extracts. This strong activity suggests that the extracts may be particularly effective against coagulase-negative staphylococci, which are common opportunistic pathogens associated with biofilm-related infections. Similarly, *S. aureus* exhibited considerable susceptibility, especially to *A. clypeolata*, which showed a twofold lower MIC and MBC compared to *A. millefolium*.

In contrast, higher concentrations of extracts were necessary for the inhibition of the growth of Gram-positive bacteria *Staphylococcus haemolyticus* and *Streptococcus haemolyticus*, indicating their moderate antimicrobial activity. The higher MBC/MIC ratios observed for these strains suggest a predominantly bacteriostatic effect at lower concentrations, with bactericidal activity occurring only at increased extract levels. As expected, Gram-negative bacteria exhibited substantially higher resistance. *Serratia marcescens* showed moderate susceptibility, whereas *P. aeruginosa* was the most resistant microorganism tested, with MIC values exceeding 75 mg/mL for both extracts. This reduced susceptibility is likely due to the intrinsic resistance mechanisms of Gram-negative bacteria, including the presence of an outer membrane, efflux pumps, and reduced membrane permeability, which limit the penetration of plant-derived antimicrobial compounds. Regarding antifungal activity, both extracts demonstrated similar and moderate efficacy against yeasts. *Meyerozyma guillermondii* was more sensitive than *C. albicans*, requiring lower MIC and MFC values for growth inhibition.

Antimicrobial activity of the extracts could be closely connected to their phytochemical composition and the presence of compounds such as phenolic acids, flavonoids, or terpenoids. These classes of compounds are known to disrupt microbial cell membranes, interfere with enzyme activity, and impair nucleic acid synthesis.

For example, it has been shown that extracts containing cosmosiin, which is represented in the highest percentage in our extracts (Section 3.3), demonstrate inhibitory effects against bacteria like *S. aureus* and yeast such as *C. albicans*, with inhibitory strength varying according to the origin and composition of the extract. According to scientific data, in addition to cosmosiin, cynaroside, also highly present in both extracts (Section 3.3), possesses significant antimicrobial properties, showing activity against various bacteria (*Escherichia coli*, *P. aeruginosa*, *S. aureus*, *Streptococcus pyogenes*, and *Micrococcus luteus*), yeast (*C. albicans*, *Candida lusitaniae*, *Saccharomyces cerevisiae*, and *S. carlsbergensis*), and molds (*Aspergillus niger*, *Penicillium oxalicum*, *Mucor mucedo*, and *Cladosporium cucumerinum*). Cynaroside exhibits its antimicrobial activity often by reducing biofilm formation, increasing drug susceptibility, or generating ROS, making it a promising natural compound for combating microbial infections and antibiotic resistance [108].

The antimicrobial activity of extracts is influenced not only by the activity of the dominant components, their structural configuration, and their functional groups, but also by complex synergistic interactions involving both major and minor compounds within the mixture. According to research by Pepeljnjak et al. [109], hyperoside shows particular antimicrobial activity against *S. aureus*, *S. epidermitis*, *S. pyogenes*, *Baccilus subtilis*, *Enterococcus faecalis*, *E. coli*, *P. aeruginosa*, *Klebsiella pneumoniae*, *Proteus mirabilis*, *Neisseria gonorrhoeae*, and *C. albicans* strains. Given these results, the extract with the aforementioned component at sufficient concentration can be used in pharmaceutical and non-pharmaceutical applications, for example, cosmetic or hygiene products [109].

In addition to hyperoside, rutin, a natural flavonoid somewhat more abundant in the *A. millefolium* extract, possesses significant antimicrobial activity against various bacteria, including Gram-positive strains (*S. aureus*, *E. coli*, *B. subtilis*, and *B. cereus*) and Gram-negative strains (*P. aeruginosa*, *Proteus vulgaris*, *Shigella sonnei*, and *K. pneumoniae*). Its mechanisms involve disrupting bacterial cell membranes, inhibiting key enzymes like DNA isomerase, interfering with biofilm formation, and potentially enhancing the efficacy of traditional antibiotics through synergistic effects, showing promise for food preservation and novel anti-infective therapies [110]. Nano-formulations of rutin have shown effectiveness in promoting infected wound healing in vivo. Its broad activity and ability to overcome antibiotic resistance make it a candidate for developing new anti-infective agents, often through nano-delivery systems [111].

The synergistic activity of all components, even those present in trace amounts (such as quercetin, known for significant antimicrobial activity), certainly contributes to the various biological activities, including antimicrobial effect, of both extracts and their potential application in the treatment of skin infections.

## 4. Conclusions

This comparative study demonstrates that both *Achillea millefolium* and the Balkan endemic *Achillea clypeolata* are rich sources of bioactive phenolic compounds and exhibit pronounced anti-inflammatory, antioxidant, enzyme-inhibitory, and antimicrobial activities. *A. millefolium* showed greater protective effects in the heat-induced hemolysis model, while both species exhibited comparable efficacy under osmotic stress conditions. Both extracts demonstrated concentration-dependent inhibition of skin-aging-related enzymes and effectively reduced AAPH-induced ROS levels at lower concentrations, although diminished effects were observed at higher doses. Moderate cytotoxicity was detected, with *A. clypeolata* exhibiting somewhat stronger effects at higher concentrations. These findings substantiate the traditional medicinal use of *Achillea* species and identify *A. clypeolata* as a promising candidate for further investigation and potential development of natural dermatological and pharmaceutical products. The lack of comprehensive data on the natural resources and cultivation of *A. clypeolata* highlights a significant knowledge gap that warrants further research.

## Figures and Tables

**Table 1:** Identification of compounds in *A. millefolium* (AM) and *A. clypeolata* (AC) extracts by UHPLC-MS/MS.

№	Rt (min)	Compound Name	Molecular Formula	[M-H], *m/z*^−^	Δ, ppm	MS/MS Fragments	**AM**	**AC**
1	0.9	D-Mannitol	C_6_H_13_O_6_	181.0710	−3.96	181, 101, 89, **71**, 59	+	+
2	0.91	Gluconic acid	C_6_H_11_O_7_	195.0504	−3.21	195, 129, **75**, 59	+	+
3	0.91	Sucrose	C_12_H_21_O_11_	341.1091	0.4	341, 179, 119, 89, 71, **59**	+	+
4	0.92	Quinic acid *	C_7_H_11_O_6_	191.0555	−3.46	**191**	+	+
5	0.93	D-Fructose	C_6_H_11_O_6_	179.0553	−4.29	179, 161, 99, **87**	+	+
6	0.96	Malic acid	C_4_H_5_O_5_	133.0554	−3.86	133, **115**, 71	+	+
7	1	Dihydroxybenzoic acid *O*- hexoside	C_13_H_15_O_9_	315.0783	2.19	315, 153, 152, 109, **108**	+	+
8	1.39	Hydroxy-methoxybenzoic acid *O*-hexoside	C_14_H_17_O_9_	329.0884	1.93	329, **167,** 152, 123, 108	+	+
9	1.45	Dihydroxybenzoic acid *O*- hexoside	C_13_H_15_O_9_	315.0730	2.58	315, 153, 152, 109, **108**	+	+
10	1.56	Neochlorogenic acid (3-*O*-caffeoylquinic acid) *	C_16_H_17_O_9_	353.0885	1.88	353, **191**, 179, 135	+	+
11	1.61	Syringic acid *O*-hexoside	C_15_H_19_O_10_	359.0990	1.75	359, **197**, 182, 153, 138, 123	+	+
12	2.17	6,7-Dihydroxycoumarin-6-*O*-glucoside (esculin)	C_15_H_15_O_9_	339.0726	1.23	339, **177**, 133	+	+
13	2.77	Dihydroxybenzoic acid *O*-pentoside	C_12_H_13_O_8_	285.0624	2.87	285, 153, 152, 109, **108**	+	+
14	2.78	4-Hydroxybenzoic acid *	C_7_H_5_O_3_	137.0234	−3.71	137, **93**	+	+
15	2.94	Chlorogenic acid (5-*O*-caffeoylquinic acid) *	C_16_H_17_O_9_	353.0885	1.88	353, **191**	+	+
16	2.99	Gentisinic acid (2,5-Dihydroxybenzoic acid)	C_7_H_5_O_4_	153.0185	−2.09	153, **109**, 108	+	-
17	3.9	Caffeic acid *	C_9_H_7_O_4_	179.0347	−1.84	179, **135**, 91	+	+
18	5.06	*5-O*-*p*-Coumaroylquinic acid	C_16_H_17_O_8_	337.0937	2.28	337, **191**, 173	+	+
19	5.66	12:4 3O fatty acyl hexoside	C_18_H_29_O_9_	387.1672	2.29	387, 207, 163, **59**	+	+
20	5.94	1,3-Dicaffeoylquinic acid *	C_25_H_23_O_12_	515.1203	1.55	353, **191**, 179, 135	+	-
21	6.39	Apigenin-6,8-di-*C*-glucoside	C_27_H_29_O_15_	593.1538	4.31	**593**, 473, 383, 353, 325, 297, 193, 161	+	+
22	6.6	5-*O*-Feruloylquinic acid	C_17_H_19_O_9_	367.1039	1.08	367, **191**	+	+
23	7.37	Luteolin-7,3′-di-*O*-glucoside	C_27_H_29_O_16_	609.1477	2.62	447, **285**	+	-
24	8.25	Luteolin-6-*C*-glucoside (homoorientin)	C_21_H_19_O_11_	447.0938	1.04	447, 357, **327**, 299, 285, 284	+	-
25	8.27	Schaftoside	C_26_H_27_O_14_	563.1416	3.68	**563**, 443, 383, 365, 353, 325, 297, 117	+	+
26	8.74	Quercetin 3-*O*-arabinoglucoside	C_26_H_27_O_16_	595.1323	3.14	595, 301, **300**, 271, 255, 179, 151	+	-
27	8.93	Quercetin 4′-*O*-glucoside	C_21_H_19_O_12_	463.0878	−0.8	463, **301**, 300	+	+
28	8.99	Isoafraxidin	C_11_H_9_O_5_	221.0453	−1.24	221, **206**, 191, 163	+	-
29	9.04	Quercetin 3-*O*-xylosylglucuronide	C_26_H_25_O_17_	609.1477	−1.77	609, **301**, 271, 227, 179, 151	+	+
30	9.28	Quercetin 3-*O*-xylosylglucoside	C_26_H_27_O_16_	595.1328	3.96	595, 301, **300**, 271, 255, 179	-	+
31	9.48	Petunidin 3-*O*-glucoside	C_22_H_21_O_12_	477.1038	−0.19	477, **315**, 300, 271	+	-
32	9.76	Apigenin 8-*C*-glucoside (Vitexin)	C_21_H_19_O_10_	431.0995	2.73	431,341, **311**, 283, 117	+	-
33	9.87	Rutin *	C_27_H_29_O_16_	609.1464	0.42	609, 301, **300**, 271, 255, 179, 151	+	+
34	9.94	Hyperoside *	C_21_H_19_O_12_	463.0878	−0.93	463, **301**, 300, 179, 151	+	+
35	9.95	Miquelianin *	C_21_H_17_O_13_	477.0689	3.03	477, **301**, 300	+	+
36	10.2	Isoquercetin *	C_21_H_19_O_12_	463.0890	1.77	463, 301, **300**, 271, 255, 179, 151	+	+
37	10.52	Luteolin 7-*O*-glucuronide *	C_21_H_17_O_12_	461.0733	1.57	461, **285**, 175	+	-
38	10.57	Luteolin 7-*O*-rutinoside	C_27_H_29_O_15_	593.1527	2.56	593, **285**, 269, 227	+	-
39	10.68	Luteolin 7-*O*-glucoside (Cynaroside) *	C_21_H_19_O_11_	447.0943	2.34	447, **285**, 284	+	+
40	11.41	Dicaffeoylquinic acid isomer	C_25_H_23_O_12_	515.1203	1.55	353, 191, 179, **173**, 161, 135	+	+
41	11.45	Patuletin xyloside	C_21_H_19_O_12_	463.0886	0.78	463, **331**, 316, 301, 287, 271	-	+
42	11.46	Quercetin-3-*O*-glucoside-6″-acetate	C_23_H_21_O_13_	505.0993	1.07	505, 301, **300**, 271, 255, 179, 151	+	-
43	11.81	Isoverbascoside	C_29_H_35_O_15_	623.1995	2.12	623, **161**, 133	+	-
44	11.83	Kaempfeol 3-*O*-rutinoside *	C_27_H_29_O_15_	593.1530	2.97	593, **285**, 284, 255, 227	+	+
45	12	3,4-Dicaffeoylquinic acid *	C_25_H_23_O_12_	515.1203	1.55	515, 353, 191, 179, **173**, 161, 135	+	+
46	12.05	Avicularin *	C_20_H_17_O_11_	433.0785	1.98	433, **301**, 300, 271, 255	-	+
47	12.11	Kaempferol-3-*O*-glucuronide	C_21_H_17_O_12_	461.0736	2.36	461, **285**, 257, 229	+	-
48	12.22	Kaempferol-3-*O*-glucoside *	C_21_H_19_O_11_	447.0940	1.59	447, 285, 284, **255**, 227	+	+
49	12.25	1,5-Dicaffeoylquinic acid *	C_25_H_23_O_12_	515.1203	1.55	353, **191**, 179, 135	+	+
50	12.43	3,5-Dicaffeoylquinic acid *	C_25_H_23_O_12_	515.1203	1.55	353, **191**, 179, 135	+	+
51	12.45	Isorhamnetin 3-*O*-rutinoside (Narcissoside) *	C_28_H_31_O_16_	623.1635	2.82	623, **315**, 314, 301, 300, 271	+	+
52	12.55	Rhoifolin (Apigenin 7-*O*-neohesperidoside)	C_27_H_29_O_14_	577.1575	2.07	577, **269**	+	-
53	12.72	Luteolin-3′-*O*-glucuronide or Luteolin-4′-*O*-glucuronide	C_21_H_17_O_12_	461.0735	2.17	461, **285,** 175	+	+
54	12.76	Dicaffeoylquinic acid isomer	C_25_H_23_O_12_	515.1203	1.55	353, **191**, 179, 135	+	+
55	12.79	Luteolin 4′-*O*-glucoside or Luteolin 3′-*O*-glucoside	C_21_H_19_O_11_	447.0945	1.72	447, **285**	+	-
56	12.9	Azelaic acid	C_9_H_15_O_4_	187.0971	−2.62	187, **125**, 97	+	+
57	12.91	Isorhamnetin 3-*O*-glucoside	C_22_H_21_O_12_	477.1036	−0.44	477, 315, **314**, 300, 285, 271, 243	+	+
58	12.93	Apigenin 7-*O*-glucoside *	C_21_H_19_O_10_	431.0992	1.81	431, **268**	+	+
59	12.99	Apigenin 7-*O*-glucuronide	C_21_H_17_O_11_	445.0783	1.44	445, **269**	+	+
60	13.8	Diosmetin 7-*O*-rutinoside	C_28_H_31_O_15_	607.1683	2.46	607, **299**, 284	+	-
61	13.81	Chrysoeriol 7-*O*-glucoside	C_22_H_21_O_11_	461.1099	2.08	461, 298, 283, **255**	+	+
62	13.83	Hispidulin glucuronide	C_22_H_19_O_12_	475.0891	1.79	475, **299,** 284, 256	+	-
63	13.92	4,5-Dicaffeoylquinic acid *	C_25_H_23_O_12_	515.1203	1.55	353, **191**, 179, 173, 135	+	+
64	16.35	Eriodictyol	C_15_H_11_O_6_	287.0557	−1.35	287, 151, **135**, 107	+	-
65	17.21	Quercetin *	C_16_H_11_O_6_	301.0359	1.7	**301**, 229, 179, 151, 121	+	+
66	17.26	Luteolin *	C_15_H_9_O_7_	285.0409	1.41	**285**, 133	+	+
67	19.07	3-Methylquercetin	C_16_H_11_O_7_	315.0517	2.23	315, **300**, 271, 255, 227	+	+
68	19.65	6,8-Dimethoxy-5,7,3,’4′-tetrahydroxyflavone	C_17_H_13_O_8_	345.0620	1.04	345, **330**, 315, 287	+	+
69	19.65	3,4,5-Tricaffeoylquinic acid	C_34_H_29_O_15_	677.1529	2.51	515, 353, 335, 191, 179, 173, 161, **135**	+	+
70	20.49	Apigenin *	C_15_H_9_O_5_	269.0460	1.65	**269**, 117	+	+
71	21.26	Diosmetin	C_16_H_11_O_6_	299.0567	1.87	299, **284**, 256	-	+
72	21.51	Chrysoeriol	C_16_H_11_O_6_	299.0565	1.26	299, **284**, 256	+	+
73	22.19	Isorhamnetin	C_16_H_11_O_7_	315.0517	2.23	**315**, 300, 271, 255, 151, 107	-	+
74	22.91	Isokaempferide	C_16_H_11_O_6_	299.0566	1.58	299, **284**, 255, 227	-	+
75	23.58	Cirsiliol	C_17_H_13_O_7_	329.0672	1.59	**329**, 314, 299, 285, 271, 243, 150, 109	+	+
76	23.83	Trihydroxyoctadecadienoic acid	C_18_H_31_O_5_	327.2185	2.56	**327**, 229, 211	+	+
77	23.96	Jaceosidin	C_17_H_13_O_7_	329.0676	2.8	329, 314, **299**, 271, 227, 150	-	+
78	24.28	Jaceidin	C_18_H_15_O_8_	359.0781	2.46	359, **344**, 329, 314, 301, 286, 202	+	+
79	24.61	Centaureidin	C_18_H_15_O_8_	359.0778	1.53	359, **344**, 329, 314, 301, 286, 258, 202	+	+
80	26.23	Trihydroxyoctadecenoic acid	C_18_H_33_O_5_	329.2340	1.92	**329**, 211, 171	+	+
81	26.43	Pectolinarigenin	C_17_H_13_O_6_	313.0727	2.88	313, 298, 297, **283**, 269, 255, 227, 183, 163, 135, 117	-	+
82	27.54	Eupatilin	C_18_H_15_O_7_	343.0830	1.9	343, **328**, 313, 298, 285, 270, 242, 186	-	+
83	27.75	Chrysosplenetin	C_19_H_17_O_8_	373.0935	1.65	373, 358, **343**, 328, 300, 285, 257, 229	+	+
84	27.83	Acacetin	C_16_H_11_O_5_	283.0617	1.91	283, **268**, 240, 239, 212, 151, 117, 107	+	+
85	28.41	Velutin (5,4′-Dihydroxy-7,3′-dimethoxyflavone)	C_17_H_13_O_6_	313.0724	2.2	313, 298, 283, **255**, 211, 183, 117	-	+
86	28.59	Santin	C_18_H_15_O_7_	343.0828	1.28	343, **328**, 313, 298, 285, 270, 242, 186	+	+

* The identity of the compounds was confirmed with authentic samples. Numbers in bold represent base peaks.

**Table 2:** Content (mg/g dry extract) of caffeoylquinic acids in *Achillea millefolium* and *A. clypeolata* extracts.

Compound	*A. millefolium*	*A. clypeolata*
5-Caffeoylquinic acid (chlorogenic acid)	21.49 ± 0.30 ^a^	13.80 ± 0.19 ^b^
3,4-Dicaffeoylquinic acid	2.68 ± 0.05 ^a^	1.95 ± 0.02 ^b^
3,5-Dicaffeoylquinic acid	23.86 ± 0.32 ^a^	15.07 ± 0.11 ^b^
1,5-Dicaffeoylquinic acid	7.59 ± 0.18 ^a^	4.16 ± 0.09 ^b^
4,5-Dicaffeoylquinic acid	7.95 ± 0.15 ^a^	4.60 ± 0.10 ^b^

The results are expressed as mean ± SD (standard deviation) from three measurements; The different letters in rows indicate a significant difference (*p* < 0.05, one-way ANOVA, Duncan’s post hoc test).

**Table 3:** Content (mg/g dry extract) of flavonoid compounds in *Achillea millefolium* and *A. clypeolata* extracts.

Compound	*A. millefolium*	*A. clypeolata*
Rutin	2.44 ± 0.04 ^a^	1.71 ± 0.03 ^b^
Hyperoside	3.50 ± 0.06 ^a^	1.72 ± 0.03 ^b^
Miquelianin	0.06 ± 0.00 ^a^	0.02 ± 0.00 ^b^
Narcissoside	1.25 ± 0.02 ^b^	2.50 ± 0.05 ^a^
Isorhamnetin	nd *	0.31 ± 0.01
Cosmosiin	7.09 ± 0.15 ^a^	4.19 ± 0.11 ^b^
Cynaroside	6.01 ± 0.13 ^a^	4.21 ± 0.09 ^b^
Quercetin	0.08 ± 0.00 ^a^	0.01 ± 0.00 ^b^
Nicotiflorin	0.33 ± 0.01 ^b^	2.48 ± 0.03 ^a^

* not detected; the results are expressed as mean ± SD (standard deviation) from three measurements. The different letters in rows indicate a significant difference (*p* < 0.05, one-way ANOVA, Duncan’s post hoc test).

**Table 4:** The inhibition activity of *Achillea millefolium* and *A. clypeolata* leaf ethanolic extracts and positive controls against skin-related enzymes, expressed as IC_50_ (µg/mL).

	IC_50_ Value ± SD (µg/mL)			
Samples	Collagenase	Elastase	Hyaluronidase	Tyrosinase
*A. millefolium*	413.30 ± 19.12 ^b^	1432.82 ± 93.21 ^a^	328.76 ± 20.28 ^b^	638.93 ± 3.06 ^b^
*A. clypeolata*	646.45 ± 16.76 ^a^	1501.66 ± 52.86 ^a^	620.60 ± 42.35 ^a^	857.87 ± 70.10 ^a^
Positive controls				
EDTA	33.43 ± 0.55 ^c^	-	-	-
EGCG	-	55.52 ± 1.93 ^b^	-	-
Oleanolic acid	-	48.41 ± 1.08 ^b^	75.76 ± 2.52 ^c^	-
Ascorbic acid	-	-	45.80 ± 1.69 ^c^	-
Kojic acid	-	-	-	64.81 ± 2.17 ^c^

For each assay, extracts and positive controls were treated as separate groups; mean values with different superscripts differ significantly (one-way ANOVA, Tukey’s post hoc; *p* < 0.05). The superscript letters indicate the values in descending order, with (a) representing the highest value. EDTA—ethylenediaminetetraacetic acid, EGCG—epigallocatechin gallate.

**Table 5:** Radical scavenging activity of *Achillea millefolium* and *Achillea clypeolata* extracts.

Sample	IC_50 ABTS_ * (μg/mL)	IC_50 DPPH_ * (μg/mL)
*Achillea millefolium* extract	22.5 ± 1.7 ^c^ **	381.93 ± 15.6 ^c^
*Achillea clypeolata* extract	18.3 ± 0.9 ^b^	303.68 ± 33.12 ^b^
Control (ascorbic acid)	2.16 ± 0.18 ^a^	31.49 ± 1.03 ^a^

* IC_50_, the sample concentration neutralizing 50% of free radicals; ** Different superscript letters in each column represent statistically significant differences (*p* < 0.05, *n* = 3, one-way analysis of variance followed by Duncan’s post hoc test).

**Table 6:** Antimicrobial activity of the *Achillea millefolium* and *A. clypeolata* extracts on the most common skin pathogens.

Microorganism	*A. millefolium* Extract		*A. clypeolata* Extract	
	MIC * (mg/mL)	MBC/MFC (mg/mL)	MIC (mg/mL)	MBC/MFC (mg/mL)
*Staphylococcus epidermidis*	0.395	1.5827	0.397	1.588
*Staphylococcus haemolyticus*	3.165	6.3308	3.177	6.354
*Staphylococcus aureus*	1.58	6.3308	0.794	1.588
*Streptococcus haemolyticus*	3.165	12.66	6.354	12.708
*Serratia marcescens*	12.66	50.647	25.417	50.835
*Pseudomonas aeruginosa*	75.97	113.962	114.38	127.087
*Meyerozyma guillermondii*	3.165	6.33	3.177	6.354
*Candida albicans*	6.33	12.6617	6.354	12.708

* MIC (minimal inhibitory concentration) and MBC/MFC (minimal bactericidal/fungicidal concentration) are expressed in mg/mL; the “stricter criteria” rule was applied, which is common in antimicrobial assays (*n* = 3, and the highest obtained value was taken as MIC and MBC/MFC).

## Data Availability

Dataset available on request from the authors.
